# Does the antidiabetic drug metformin affect embryo development and the health of brown trout (*Salmo trutta* f. *fario*)?

**DOI:** 10.1186/s12302-018-0179-4

**Published:** 2018-12-07

**Authors:** Stefanie Jacob, Andreas Dötsch, Sarah Knoll, Heinz-R. Köhler, Eike Rogall, Dominic Stoll, Selina Tisler, Carolin Huhn, Thomas Schwartz, Christian Zwiener, Rita Triebskorn

**Affiliations:** 1 Animal Physiological Ecology, University of Tübingen, Auf der Morgenstelle 5, 72076 Tübingen, Germany; 2 Department of Physiology and Biochemistry of Nutrition, Max Rubner-Institut, Haid-und-Neu-Straße 9, 76131 Karlsruhe, Germany; 3 Department of Safety and Quality of Fruit and Vegetables, Max Rubner-Institut, Haid-und-Neu-Straße 9, 76131 Karlsruhe, Germany; 4 Effect-based Environmental Analysis, University of Tübingen, Auf der Morgenstelle 18, 72076 Tübingen, Germany; 5 Interface Microbiology, Karlsruhe Institute of Technology, Hermann-von-Helmholtz-Platz 1, 76344 Eggenstein-Leopoldshafen, Germany; 6 Environmental Analytical Chemistry, University of Tübingen, Hölderlinstr.12, 72074 Tübingen, Germany; 7 Steinbeis Transfer Center for Ecotoxicology and Ecophysiology, Blumenstr. 13, 72108 Rottenburg, Germany

**Keywords:** Pharmaceutical, Salmonid, Glycogen, Body weight, Microbiome

## Abstract

**Background:**

Due to the rising number of type 2 diabetes patients, the antidiabetic drug, metformin is currently among those pharmaceuticals with the highest consumption rates worldwide. Via sewage-treatment plants, metformin enters surface waters where it is frequently detected in low concentrations (µg/L). Since possible adverse effects of this substance in aquatic organisms have been insufficiently explored to date, the aim of this study was to investigate the impact of metformin on health and development in brown trout (*Salmo trutta* f. *fario*) and its microbiome.

**Results:**

Brown trout embryos were exposed to 0, 1, 10, 100 and 1000 µg/L metformin over a period from 48 days post fertilisation (dpf) until 8 weeks post-yolk sac consumption at 7 °C (156 dpf) and 11 °C (143 dpf). Chemical analyses in tissues of exposed fish showed the concentration-dependent presence of metformin in the larvae. Mortality, embryonic development, body length, liver tissue integrity, stress protein levels and swimming behaviour were not influenced. However, compared to the controls, the amount of hepatic glycogen was higher in larvae exposed to metformin, especially in fish exposed to the lowest metformin concentration of 1 µg/L, which is environmentally relevant. At higher metformin concentrations, the glycogen content in the liver showed a high variability, especially for larvae exposed to 1000 µg/L metformin. Furthermore, the body weight of fish exposed to 10 and 100 µg/L metformin at 7 °C and to 1 µg/L metformin at 11 °C was decreased compared with the respective controls. The results of the microbiome analyses indicated a shift in the bacteria distribution in fish exposed to 1 and 10 µg/L metformin at 7 °C and to 100 µg/L metformin at 11 °C, leading to an increase of Proteobacteria and a reduction of Firmicutes and Actinobacteria.

**Conclusions:**

Overall, weight reduction and the increased glycogen content belong to the described pharmaceutical effects of the drug in humans, but this study showed that they also occur in brown trout larvae. The impact of a shift in the intestinal microbiome caused by metformin on the immune system and vitality of the host organism should be the subject of further research before assessing the environmental relevance of the pharmaceutical.

**Electronic supplementary material:**

The online version of this article (10.1186/s12302-018-0179-4) contains supplementary material, which is available to authorized users.

## Introduction

In recent decades, the rising number of micropollutants like industrial chemicals, pharmaceuticals, personal care products and pesticides detected in the water cycle has become a topic of interest in the public and the scientific community [3, 33, 65]. At the same time, the analytical instruments and methods were improved, which made the detection of these substances in the picogram to microgram per litre range possible [1, 3]. In particular, human pharmaceuticals are constantly discharged into water bodies, leading to the continuous exposure of aquatic organisms. Since they have a specific mode of action and are made to exert an effect in humans, it is likely that they have an effect in aquatic non-target organisms [12, 15]. The antidiabetic drug metformin belongs to the group of most frequently prescribed pharmaceuticals worldwide [24, 32, 34, 70]. In 2015, 590 million defined daily doses (DDD) of this drug were prescribed in Germany [64], which resulted in a total prescribed amount of 1180 t, assuming a DDD of 2 g. The human body excretes the pharmaceutical without any metabolisation [5, 60]. Although the elimination rates of metformin are > 90% in sewage-treatment plants, mostly due to microbial action, this compound occurs in surface waters at concentrations up to 3 µg/L [61].

The therapeutic action of metformin in type 2 diabetes patients is the reduction of blood glucose levels by (1) the inhibition of hepatic gluconeogenesis and glycogenolysis and (2) the activation of glucose uptake into cells [2, 30, 47, 75]. Despite the fact that numerous studies have addressed the mechanism of action of this drug, it is not fully understood to date. Gluconeogenesis—a very energy-demanding process—is probably reduced by the inhibition of the mitochondrial respiration, which leads to a decrease in the ATP/ADP ratio [18, 21, 22, 29]. The improved glucose uptake in cells is most likely caused by an enhanced glucose transporter capacity and the translocation of these transporters into the cell membrane [25, 30, 43, 75]. Although it is not known whether the same mechanisms could be found in fish, it is likely that metformin could exert the same effects in their mitochondria [26].

In addition to interactions with carbohydrate metabolism, an indirect impact on other metabolic pathways is probable and still under investigation. In this context, it should be taken into account that, apart from its application as an antidiabetic, metformin is also used as a weight loss drug [66], for the treatment of the polycystic ovary syndrome, a disease which inhibits ovulation and evokes high levels of androgens [39, 48], and as an anti-cancer drug [42].

It has been demonstrated that metformin exerts similar therapeutic effects on non-target organisms. For example, Crago et al. [13] detected an increase of vitellogenin mRNA in juvenile fathead minnow (*Pimephales promelas*) exposed for 7 days to 1, 10 and 100 µg/L metformin. Niemuth and Klaper [50, 51] showed 40 µg/L metformin to affect several endocrine-related genes and to evoke intersexuality and a reduced size in adult male fathead minnows and the reduced fecundity of mating pairs which had been exposed from the fry stage to adulthood for about 1 year. In a further study, Niemuth et al. [49] demonstrated that a concentration of 40 µg/L metformin increased the vitellogenin mRNA in adult fathead minnow exposed for 28 days. Ussery [73] showed that metformin reduced the growth of Japanese medaka (*Oryzias latipes*) exposed to 1–100 µg/L metformin for 28 days. In contrast, in a report [19] cited by Moermond and Smit [45], a NOEC ≥ 10 mg/L metformin was derived for hatching rate, time to hatch, survival, length and weight in an early-life stage toxicity test with zebrafish (*Danio rerio*). Furthermore, several studies showed that metformin changed the composition of the intestinal microbiome in humans and mice [23, 37, 67, 77]. This is especially important, since studies found a strong correlation between the composition of the gut microbiome and the occurrence of diseases [16, 31, 41, 78]. Moreover, Piro et al. [54] demonstrated that metformin leads to a reduction of enhanced stress protein levels caused by exposure to high levels of free fatty acids in the pancreatic islets of rats.

In our study, we investigated the impact of metformin on the embryonic development and different health indicators of brown trout larvae. Possible embryotoxic effects of the drug were investigated by means of a modified fish early-life stage test with investigative endpoints including mortality, time to hatch, heart rate and growth. Further biomarkers concerning the health of brown trout larvae were the histopathology of the liver, analyses of hepatic glycogen content, stress protein levels, swimming behaviour and the intestinal microbiome. With the aim of revealing the impact of temperature on potential metformin effects, fish were exposed to four concentrations of the antidiabetic drug plus a negative control at two different temperatures (7 °C and 11 °C). Since the mean annual optimal river water temperature for brown trout has been determined to be 9 °C [28], we selected two temperatures, one lower (7 °C) and one higher (11 °C), in order to take the change of seasons into account. The exposure experiment was controlled by chemical analysis of the metformin concentration in the exposure medium. The uptake of metformin into test organisms was also characterised.

In the liver of larvae, alterations in tissue integrity were examined by histopathological studies. We also determined the glycogen content in the liver as an important parameter for the energetic status of fish, both by biochemical and histological analyses. Several studies showed that chemicals, including pharmaceuticals, can lead to the depletion of hepatic glycogen in trout as a result of increased energy demand for biotransformation [9, 63, 71].

As a general stress marker, the level of heat shock proteins with a weight of 70 kDa (Hsp70) was examined in the heads of fish to investigate whether metformin can change the Hsp70 level of brown trout. Hsp70 proteins belong to the stress protein family and support the folding of the unfolded and the refolding of damaged proteins.

As mentioned above, the effects of metformin on the intestinal microbiome are a topic of current interest [23, 77]. Therefore, we studied the microbiome composition in the gut of metformin-exposed brown trout.

In addition, the swimming behaviour of fish (total distance moved and mean velocity) was quantified because changes in the energy supply may also manifest in behavioural alterations.

Overall, our study aimed to investigate whether metformin negatively influences the vitality of brown trout and, if so, whether such health loss is related to changes in the gut microbiome or in carbohydrate metabolism.

## Materials and methods

### Test organisms

For the present study, eggs of brown trout in the eyed-ova stage (48 dpf) were obtained from a commercial fish breeder (trout breeding Lohmühle, D-72275 Alpirsbach-Ehlenbogen) whose fish breeding is listed as category I (disease-free) according to the EC Council Directive [20].

### Test substance

Metformin hydrochloride (CAS number: 1115—70—4) was purchased from Sigma-Aldrich (Steinheim, Germany; purity 99.9%; Batch number: BCBP0558V). The substance was readily soluble in water with a water solubility of 16.56 g/L [69]. The concentrations of metformin investigated in the present study refer to metformin free base (C_4_H_11_N_5_) and not the hydrochloride.

### Exposure experiments and sampling

Eyed eggs of brown trout (48 dpf, incubation temperature 8 °C) were exposed directly after purchase to five different nominal concentrations of metformin (0, 1, 10, 100, 1000 µg/L) in triplicate at 7 °C and 11 °C in climate chambers. The exposure was conducted in a semi-static system using glass aquaria with 28 test organisms per 10 L aquarium. Thus, in total, 840 individuals were investigated for their development and health. After 57 days (for larvae exposed to 11 °C) and 73 days (for larvae exposed to 7 °C), depending on the developmental status of the brown trout, half of the fish were sampled. These samplings were performed 3 weeks post-yolk sac consumption. The other half of the fish was sampled after 95 days for brown trout exposed to 11 °C and 108 days for those at 7 °C. These samplings took place 8 weeks after yolk sac consumption of the larvae. In this study, only data of the larvae sampled 8 weeks post-yolk sac consumption is presented.

Twice a week, 50% of the exposure medium was exchanged for freshly prepared medium. For the preparation of the medium, aerated, filtered tap water (iron filter, active charcoal filter, particle filter) was used. The medium in the aquaria was aerated with air stones (JBL Pro Silent Aeras Micro S2). The light/dark conditions were kept constant during the test with a 10 h/14 h—light/dark cycle. Additionally, the aquaria were shaded from direct light with black plastic foil.

During the experiment, the time to hatch and mortality were recorded every day. The heart rates of fifteen larvae from both the control and the treatment with the highest metformin concentration (1000 µg/L metformin) were counted for 20 s after 21 days of exposure for fish at 11 °C and after 37 days of exposure for fish at 7 °C, when the brown trout were in the finfold resorption phase (step 38/39 according to Killeen et al. [35]). Therefore, the brown trout larvae were transferred to vessels containing the respective test medium at the respective temperature. For the last 8 weeks of exposure, the larvae were fed every day with commercial trout food (INICIO plus from Biomar, Denmark) since their yolk sacs had already been consumed. The defined amount of food was constantly adapted according to the developmental status of the brown trout. Excess food and faeces were removed during the water exchange. Temperature, pH, oxygen content and conductivity were monitored at the beginning and end of the experiment in all treatments as well as at days 21 (11 °C) and 37 (7 °C) of the experiment for the controls and the highest concentration (details in the result chapter and Additional file 1: Paragraph 3). At the end of the experiment, the fish were euthanised with an overdose of MS 222 (1 g/L tricaine methanesulphonate buffered by NaHCO_3_) and subsequent severance of the spine. The length and weight of fish as well as possible abnormalities or injuries were recorded. Due to the small size of the test organisms, the fish were separated into two groups (details in Additional file 1: Paragraph 10, Fig. S3). From the first group, samples for histopathology (liver) and chemical tissue analysis (kidney, muscle, and head without gills) were obtained. The second group provided samples for the analyses of hepatic glycogen content (liver), stress protein levels (head) and the intestinal microbiome (gut). Samples for histological analyses were chemically fixed with glutardialdehyde. All other samples were immediately frozen in liquid nitrogen and stored at − 80 °C.

### Chemical analyses

During the experiments, water samples were taken to determine the real metformin concentrations in the test aquaria. Water samples were taken at the beginning (05.01.16) and end of the experiment (11 °C: 08.04.16, after 97 days; and 7 °C: 21.04.16, after 108 days), as well as at different time points (after 10, 41, 63 and 79 days; details in Additional file 1: Paragraph 4, Tables S9 and S10) before and after the water exchanges and stored at − 20 °C until processing. At the end of the experiment, tissue samples of muscle, kidney and head without gills were investigated to determine the internal metformin concentration in the fish.

#### Analysis of water concentrations by LC–MS

Quantitative measurements of the water samples were performed by LC-MS using a 1260 Infinity HPLC system (Agilent Technologies, Waldbronn, Germany) with a triple quadrupole mass spectrometer (QqQ-MS: 6490 Agilent Technologies Santa Clara, CA, USA). A Phenomenex LUNA 5 u HILIC 200 A column (150 × 3 mm; 5 μm particle size) was used for separation at a flow rate of 0.5 mL/min and 40 °C. Eluent A was an aqueous buffer with 15 mM ammonium formate and 0.1% formic acid (FA), while eluent B was acetonitrile (AcN) with 0.1% FA (all chemicals were purchased from Fisher Scientific, Schwerte, Germany). A gradient elution was performed: using 0–4 min 95% eluent B, decreasing to 50% eluent B within 4 min, and then being held for 6 min at 50% eluent B. After switching back to the starting conditions, the post time was 8 min. The standard metformin hydrochloride (> 98%) was purchased from Tokyo Chemical Industry (TCI, Tokyo, Japan). Individual stock solutions with a concentration of 1 g/L were prepared in a mixture of AcN and water (1:1). All working solutions of the standard for direct injection were prepared in AcN and ultrapure water (10:1). Stock and working solutions were stored in the freezer at − 20 °C, except for the aqueous isotopically labelled metformin-d6 solution (from Toronto Research Chemicals) which was stored at + 4 °C.

Samples were kept in the autosampler at 10 °C, and the injection volume was 10 µL. All samples had a composition of 90% AcN and 10% H_2_O due to dilution (dilution factor between 10 and 500). Calibration was performed between 0.1 and 10 μg/L in 90% AcN and 10% H_2_O with metformin-D6 as an internal standard. The concentration of the internal standard was 1 μg/L in all diluted samples and calibration standards. Quantification of metformin was achieved after LC separation using a 6490 triple quadrupole mass spectrometer with the positive ionisation mode. The electrospray ionisation source with an Agilent Jet Stream technology was operated under the conditions given in Additional file 1: Table S1. The recorded data were processed using the software Mass Hunter (Agilent Technologies). For quantification and confirmation, two multiple reaction monitoring (MRM) transitions were monitored for each analyte in the dynamic MRM mode. Details are given in Additional file 1: Table S2. The limit of quantification was 1 ng/L for metformin. Figures of merit for the analysis method are given in Additional file 1: Paragraph 1, Table S3.

#### Metformin analysis in tissue of brown trout larvae by CE–MS

The metformin concentration in the tissue of brown trout fry was determined by capillary electrophoresis–mass spectrometry (CE–MS). Fish samples (from the head (without the gills) to the tail fin, including the kidney and muscle, but not the liver or intestine) originating from all exposure concentrations were analysed. For each exposure group, tissue samples of 21 individuals per treatment were pooled to reach the required detection limits. After the generation of two subsamples per treatment, these were measured to determine methodological precision. For sample preparation, frozen (− 20 °C) samples were first homogenised by grinding using a mortar and pestle under liquid nitrogen. A total of 100 mg of the homogenised sample was transferred to an Eppendorf tube; metformin-d6 as an internal standard and methanol as the extraction solvent were added to make a final concentration of 291 nmol/L. The tube was vortexed for 30 s, and the analytes were extracted under sonication for 15 min. Subsequently, the sample was centrifuged at 13,000 g for 15 min. After filtration using a 45 µm PTFE filter (pore size 0.45 μm, Chromafil, Macherey-Nagel, Germany), the samples were directly analysed by CE–MS. Quantification was based on the deuterated internal standard metformin-D6 with a detection limit of 0.6 μg/L. Further details can be found in Additional file 1: Paragraph 1.

All analyses were performed using an Agilent CE 7100 interfaced to an Agilent 6550 iFunnel Q-TOF mass spectrometer (Agilent Technologies, Waldbronn, Germany and Santa Clara, CA, USA) using an electrospray ionisation source assisted by the sheath-liquid interface. The CE separations were carried out by means of an uncoated fused-silica capillary (length 80 cm, i.d. 50 µm). The background electrolyte was a mixture of 100 mM ammonium acetate and 3% glacial acetic acid in methanol. Samples were injected hydrodynamically by applying a pressure of 100 mbar for 10 s. The CE capillary was kept at 25 °C during CE runs and a voltage of + 30 kV was applied. Details on the CE–MS method are given in Additional file 1: Paragraph 1.

### Histopathological investigation

Liver samples for histological analyses (21 per treatment) were fixed in 2% glutardialdehyde (25% solution in water; Merck KGaA, Darmstadt, Germany) diluted with a cacodylate-sodium buffer (0.1 M, pH 7.6; AppliChem GmbH, Darmstadt, Germany) for at least 2 weeks. Each liver sample was the whole organ of an individual due to the small size of the fish. For histopathological analyses, fixed liver samples were washed three times for 10 min with the same buffer, dehydrated in a graded series of ethanol and infiltrated with paraffin wax (Parablast, Leica, Wetzlar, Germany) in a tissue processor (TP 1020, Leica). After paraffin embedding, samples were cut into 3 µm slices using a Leica SM 2000 R microtome. There were eight slices per slide from four different planes of the organ. One portion of the slices was stained with haematoxylin–eosin (to visualise the nuclei, cytoplasm, connective tissue and muscles), and the other with alcian blue-PAS (to visualise mucus and glycogen; technical details in Additional file 1: Paragraph 2). Analyses were conducted using a microscope (Axioskop 2, Zeiss, Oberkochen, Germany). Slides were first examined qualitatively to gain an overview and identify pathologies. In a second step, the observed pathologies were semi-quantitatively assessed and classified into one of five different categories (1: control, 2: slight reaction, 3: medium reaction, 4: strong reaction, and 5: destruction) according to the criteria published by Triebskorn et al. [72]. In a further step, all samples were analysed a second time after being blinded and randomised to avoid an observer bias. In addition, all sections were categorised according to their glycogen content (high, medium and low), again in a blinded and randomised manner. A high glycogen content was described by the dark-red staining of glycogen which can be found evenly in the whole liver. A bright-red stain and a partial spread of glycogen in the liver indicated a medium glycogen content. If almost no glycogen was visible, the sample was classified as low glycogen amount.

#### Stress protein analysis

To determine the level of the 70 kD stress protein family (Hsp70) in the fish heads (21 per treatment), the samples were homogenised with a mixture of 98% extraction buffer and 2% protease inhibitor (3 mL mixture/g sample) as described by Dieterich et al. [17]. Subsequently, the total protein content in the samples was quantified according to Bradford [8]. To assess the level of Hsp70 proteins, a standardised amount of 40 µg total protein per sample was used for analysis. Using minigel SDS-PAGE, the proteins were separated according to their weight. Subsequently, the proteins were semi-dry blotted onto a nitrocellulose membrane. A primary antibody (monoclonal α-Hsp70 IgG; Dianova Hamburg, Germany), binding specifically to Hsp70 protein, was transferred to the membrane, followed by a second antibody (peroxidase-coupled α-IgG; Jackson Immunoresearch, West Grove, PA, USA) binding to the first. Finally, the membranes were stained with 4-chloro-1-naphthol, and the optical volume (= band area × average grey scale value) was quantified relative to an internal standard (brown trout whole body homogenate).

### Glycogen analysis

For quantification of the glycogen content, the glycogen assay from Sigma-Aldrich (MAK016; Steinheim, Germany) was used. Liver samples (number of individuals per treatment in Fig. 4) were homogenised with bi-distilled water (10 µL/mg tissue) on ice. Then, the samples were heated to 97 °C for 5 min to inactivate enzymes and centrifuged (Eppendorf 5424R) for 5 min at 13,000 g at 4 °C. Before conducting the assay, the supernatants of the samples were diluted 1:15 with bi-distilled water. For the assay, 96-well plates were used. Glycogen standards (0, 0.4, 0.8, 1.2, 1.6, 1.8, anf 2.0 µg/well) and liver samples were tested in duplicate. Then, 2 µL of a hydrolysis enzyme mix was added per well. In addition to the duplicates per sample, each sample was also pipetted without the hydrolysis enzyme mix (sample blank) to determine the background signal caused by glucose in the sample. The plate was incubated for 30 min at room temperature on a shaker. After adding 50 µL of the master reaction mix (containing 46 µL development buffer, 2 µL development enzyme mix and 2 µL fluorescent peroxidase substrate) per well, the plate was covered in aluminium foil and incubated for 30 min on a shaker at room temperature. Measurements were then conducted in a photometer (BioTek Instruments ELx800G, USA) at 570 nm.

### Analyses of swimming behaviour

The swimming behaviour of the test organisms was assessed after 90 days of exposure at 11 °C and after 102 days of exposure at 7 °C. Therefore, five individuals per replicate (15 individuals per treatment) were transferred into smaller square aquaria (17 cm edge-length) containing 300 mL of the respective test medium with the respective temperature. The fish tracking system was placed in the 11 °C climate chamber and the cameras were adjusted for this set up. Therefore, brown trout larvae were recorded in the 11 °C climate chamber when exposed previously to either 11 °C or 7 °C. From each replicate of the five exposure groups, five larvae were kept in one aquarium. After acclimation for 2 min, the swimming behaviour of fish was recorded for 18 min using a digital camera (Basler acA1300–60 gm camera, 1.3 MP resolution, Basler AG, Ahrensburg, Germany). The swimming behaviour was recorded in four aquaria simultaneously and analysed with respect to the total distance moved as well as the mean velocity of each individual using EthoVision 11.5 (Noldus, Wageningen, Netherlands) software. Therefore, the centre-point of the fish was tracked. After acquisition of the data, a correction of identity swaps between the tracked fish was accomplished using the Track Editor of EthoVision.

### Intestinal microbiome

For investigations of the intestinal microbiome of the test organisms, DNA was extracted from gut mucus samples and analysed via 16S amplicon-sequencing. Prior to the preparation of DNA extraction, the guts of the fish were squeezed out and washed with 50 µL sterile water to ensure that the gut content were completely removed. This step was necessary because the mucus is closely associated with microvilli in the gut [58] and therefore more relevant for the host–symbiont relationship and host–symbiont communication than the allochthonous microbiome in the gut content. Due to the young age of the fish, the guts were too small to rinse with peptone water to extract the adherent bacteria of the mucus; therefore, the whole gut was used for analysis [57]. The limited lab capacity only allowed us to analyse two pooled samples per treatment (each pooled sample contained three guts) for the exposure at 11 °C and a single pooled sample per treatment for the exposure at 7 °C. DNA was extracted using the QIAamp DNA Mini Kit (Qiagen, Hilden, Germany). The DNA was eluted in 50 µL of DNAse-free water solution (MP Biomedicals Germany GmbH; Eschwege, Germany) and quantified via NanoDrop ND-1000 spectrophotometer. The V1–V2 region of the 16S rRNA gene was amplified using 27F and 338R primers as described previously by Camarinha-Silva et al. [10]. The forward primer contains a 6-nt barcode and a 2-nt CA-linker. Both primers comprised sequences complementary to the Illumina-specific adaptors to the 5’-ends. Amplification was performed in a total volume of 50 µL with 10× polymerase chain reaction (PCR) buffer, each containing dNTPs at a concentration of 10 mM, primers at a concentration of 0.4 µM, 1 µL of template DNA, and 0.25 µL HotStarTaq Polymerase (Qiagen, Hilden, Germany). An initial denaturation step at 96 °C for 3 min was followed by 15 cycles of the following procedure: denaturation at 95 °C for 10 s, annealing at 55 °C for 10 s, and extension at 72 °C for 45 s. One microlitre of this reaction mixture served as the template for a second PCR, which was performed under the conditions described above, but with 20 cycles, using primers designed to integrate the Illumina-multiplexing sequences and indices. Non-template controls were used. All were free of any amplification products after both PCRs. PCR amplicons were verified by agarose gel electrophoresis and quantified by means of the Qubit fluorometric quantitation assay (Invitrogen, Carlsbad, USA). Sequencing was carried out on the Illumina MiSeq system using v3 chemistry with 2 × 301 cycles. Sequences were demultiplexed using a custom Perl script and processed on the Mothur platform [62] according to the MiSeq SOP published by Kozich et al. [36]. Briefly, sequences were filtered by removing sequences with ambiguous base calls and homopolymers longer than 12 nt and aligned with the Silva database v128 [56]. Chimeric sequences were removed using the VSEARCH algorithm [59] and sequences were pre-clustered allowing for 3 mismatches. Finally, sequences were classified using Silva v128 taxonomy and operational taxonomic units (OTUs) were picked at a 97% identity level. Low-abundance OTUs were removed to reduce the number of spurious OTUs.

### Statistical analysis

Statistics were analysed using JMP 12 from SAS (Cary, NC, USA). If necessary, data were squared to achieve normal distribution and homoscedasticity. This transformation was done for the data of the biochemical glycogen analysis and the stress protein analysis at 11 °C. The analyses of survival time and time to hatch were conducted with COX-regression. The data of the semi-quantitative histological examination of the tissue and the glycogen content were checked for significance with the likelihood ratio test. All other parameters were analysed using a nested ANOVA using the replicate aquaria as a nesting factor, followed by Dunnett’s post-hoc test. For the statistical analysis of the stress protein level, the total distance moved and the mean swimming velocity at 11 °C, a Welch-ANOVA was performed since the data could not be transformed to reach homoscedasticity. In “Results” section, the statistical tests used and the *p*-values are shown. Further information (e.g. degrees of freedom and *F*-value) is given in Additional file 1: Paragraph 11, Table S15. The α-level was set to 0.05. Because we had no replicate climate chambers for 7 °C and 11 °C, we refrained from statistically comparing the results obtained at the two temperatures to avoid the problem of pseudoreplication. Because of the limited number of examined replicates, data obtained for the intestinal microbiome were not statistically checked for significance. In both cases, we restricted comparisons to qualitative aspects.

## Credibility of data

Details about the fulfilment of the criteria for reporting and evaluation of ecotoxicity data (CRED) according to Moermond et al. [44] are given in Additional file 1: Paragraph 9, Table S13. CRED helps to improve the reproducibility, consistency and transparency of reliability and relevance criteria of ecotoxicity studies [44].

## Results

### Water quality parameters

Temperature, pH, oxygen content and conductivity were measured at the beginning and end of the experiments (for 7 °C: mean temp. = 7.16 ± 0.30 °C, mean pH = 8.05 ± 0.33, mean oxygen = 10.54 ± 0.16 mg/L, mean conduct. = 410.43 ± 20.29 µS/cm; for 11 °C: mean temp. = 10.74 ± 0.23 °C, mean pH = 8.03 ± 0.13, mean oxygen = 9.75 ± 0.15 mg/L, mean conduct. = 431.03 ± 8.51 µS/cm). Moreover, the water quality parameters were measured when the heart rate was recorded (for the controls and the highest metformin concentration). Further details of the water quality parameters are given in Additional file 1: Paragraph 3.

### Exposure conditions

The quantification of metformin in the test medium revealed a good accordance between the nominal and the measured metformin concentrations in our experiment; the recovery was above 80%. Therefore, we refer to the nominal concentrations throughout the entire study. The real concentrations were shown to be slightly lower than the nominal concentrations (Table 1).

**Table 1:** Measured metformin concentrations in medium and tissue, mortality, biometric data, biochemical, developmental and behavioural parameters of brown trout larvae exposed to metformin

Treatment (nominal metformin concentrations (µg/L))	7 °C					11 °C				
	0	1	10	100	1000	0	1	10	100	1000
Measured metformin water concentrations (µg/L)	< LoQ	0.8 ± 0.2	8.1 ± 0.5	97.0 ± 11.4	950.9 ± 108.1	< LoQ	0.8 ± 0.2	8.6 ± 0.9	98.7 ± 5.2	921.1 ± 106.4
Metformin tissue concentration (ng/g) wet weight	< LoQ	< LoQ	4	42	56	< LoQ	< LoQ	5	42	234
Mortality (%)	2.0 ± 1.6	0.7 ± 0.9	1.3 ± 0.9	1.3 ± 0.9	1.3 ± 0.9	3.3 ± 2.5	1.3 ± 0.9	4.0 ± 2.8	2.7 ± 0.9	0.7 ± 0.9
Heart rate (bpm)	77.4 ± 7.0	n.e.	n.e.	n.e.	74.4 ± 6.2	98.2 ± 12.2	n.e.	n.e.	n.e.	100.6 ± 10.8
Mean time to hatch (dpf)	54.5 ± 2.5	54.8 ± 2.7	53.9 ± 2.8	54.1 ± 3.0	54.6 ± 2.8	51.4 ± 1.2	51.4 ± 1.4	51.4 ± 1.4	51.0 ± 1.1	51.2 ± 1.3
Body weight (g)	0.545 ± 0.09	0.506 ± 0.09	0.480 ± 0.09*	0.491 ± 0.09*	0.566 ± 0.09	0.959 ± 0.26	0.769 ± 0.29*	0.902 ± 0.25	0.885 ± 0.23	0.829 ± 0.26
Body length (cm)	3.5 ± 0.2	3.4 ± 0.2	3.5 ± 0.2	3.5 ± 0.2	3.6 ± 0.2	4.2 ± 0.4	4.2 ± 0.5	4.2 ± 0.4	4.3 ± 0.4	4.2 ± 0.4
Hepatic glycogen content (µg/µL)	9.75 ± 3.94	14.75 ± 1.77	14.19 ± 3.84	13.38 ± 2.97	12.07 ± 5.65	10.07 ± 4.55	15.28 ± 2.13*	13.25 ± 2.41	11.04 ± 3.60	11.78 ± 3.37
Hsp70 level (rel. grey value)	1.05 ± 0.24	0.99 ± 0.13	1.07 ± 0.20	1.03 ± 0.19	0.97 ± 0.19	1.04 ± 0.27	0.95 ± 0.25	0.97 ± 0.15	0.87 ± 0.23	0.88 ± 0.21
Total distance moved (cm)	2119.49 ± 664.19	2321.80 ± 1020.74	2407.21 ± 1034.18	2218.53 ± 1087.29	2321.57 ± 695.89	1414.14 ± 889.91	1337.55 ± 388.98	1461.24 ± 515.14	1464.91 ± 582.54	1437.23 ± 515.47
Mean velocity (cm/s)	1.96 ± 0.62	2.15 ± 0.95	2.23 ± 0.96	2.06 ± 1.01	2.15 ± 0.64	1.31 ± 0.82	1.24 ± 0.36	1.35 ± 0.48	1.36 ± 0.54	1.33 ± 0.48

All data except for the analytical tissue measurements are shown as arithmetic mean ± standard deviation (SD). For the measurement of the tissue concentration, details of the method variation are given in Additional file 1: Table S4. Heart rate was only evaluated for the negative control and the highest metformin concentration, so no evaluation took place for 1, 10 and 100 µg/L. Asterisks (*) indicate significant differences compared to the respective control at the *p* = 0.05 level

*LoQ* limit of quantification, *n.e.* not evaluated, *bpm* beats per minute, *dpf* days post fertilisation

**Fig. 1:**
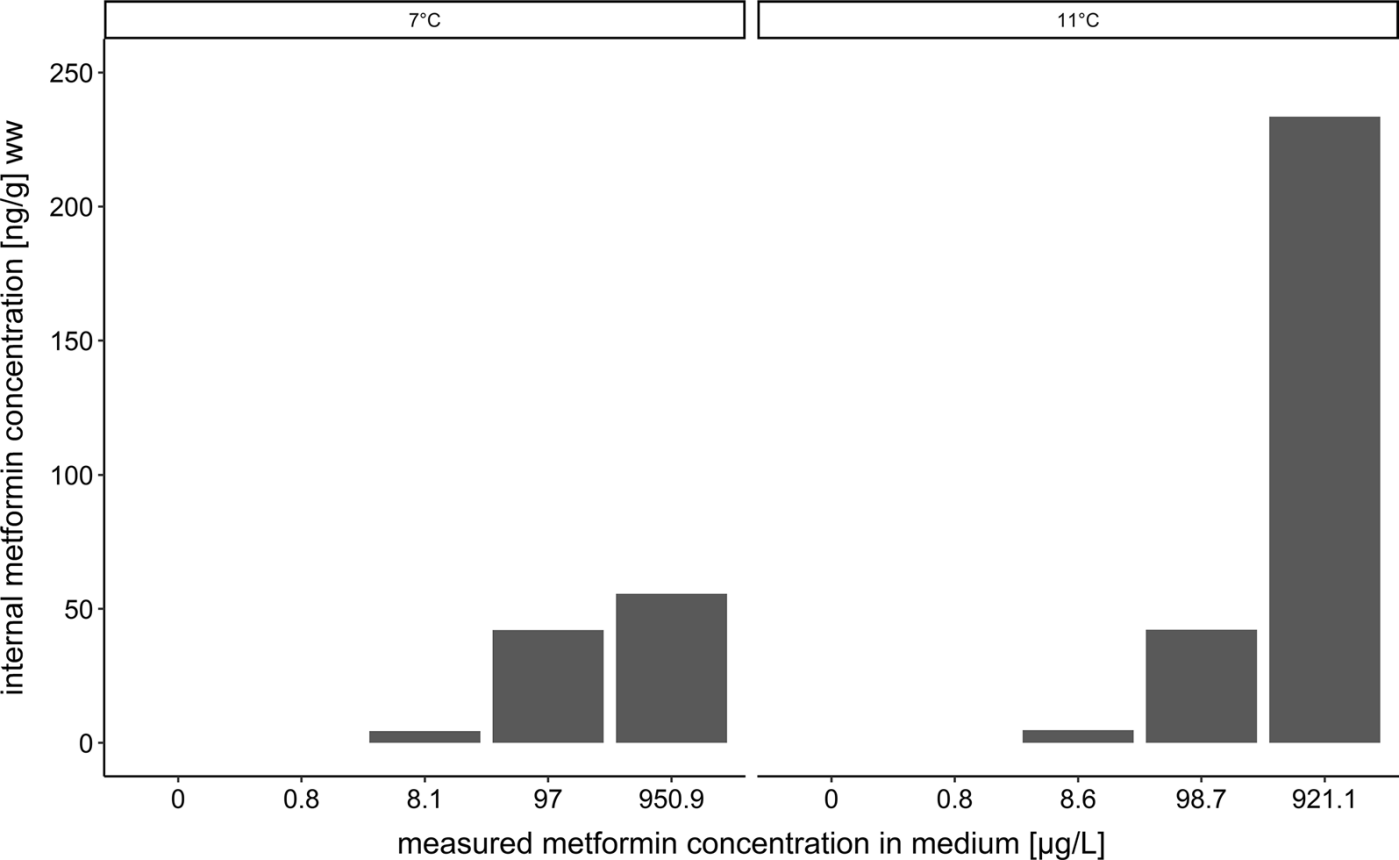
Internal metformin concentration in brown trout larvae after 95 days of exposure at 11 °C and 108 days at 7 °C (ng/g) wet weight vs. measured concentration in water (μg/L). At nominal water concentrations of 0 and 1 µg/L metformin, the internal metformin concentrations were below the limit of detection

In Fig. 1, the tissue concentrations of metformin in brown trout larvae are plotted against the measured exposure concentrations (for data, also see Table 1). Internal metformin concentrations were in the ng/g range. At nominal concentrations of the exposure medium below 10 µg/L, tissue metformin concentrations were below the limit of detection. The measured metformin tissue concentrations of brown trout larvae exposed to 1000 µg/L were higher at 11 °C compared to 7 °C. For the test organisms exposed to 10 and 100 µg/L, the metformin tissue concentrations did not differ between the temperatures in the respective exposure groups.

### Investigated developmental and health parameters

In all exposure groups, mortality was between 0.67 and 4.0% (Table 1) (COX-regression: 7 °C: *p* = 1; 11 °C: *p* = 1). Mean time to hatch did not differ between the exposure groups, at either 7 °C or 11 °C (COX-regression: 7 °C: *p* = 0.4966; 11 °C: *p* = 0.5853). Concerning the temperature, the mean time until hatching was about 4 days earlier at 11 °C compared to 7 °C for controls. The heart rate did not reveal any differences between the negative control and the highest metformin concentration (nested ANOVA: 7 °C: *p* = 0.1590; 11 °C: *p* = 0.5905) at both temperatures. However, the heart rate of control fish at 11 °C was about 21 beats/min higher than that in control fish at 7 °C.

### Body length and weight

For body weight, the statistical analysis revealed a difference between treatments at 7 °C and 11 °C (nested ANOVA: 7 °C: *p* < 0.0001; 11 °C: *p* = 0.0137) with weights of brown trout exposed to metformin concentrations of 10 and 100 µg/L at 7 °C being lower compared with the negative control (Dunnett’s test: (0 µg/L|10 µg/L): *p* = 0.0072, (0 µg/L|100 µg/L): *p* = 0.0353). Also, fish exposed to 1 µg/L metformin at 11 °C weighed less than the negative control (Dunnett’s Test: (0 µg/L|1 µg/L): *p* = 0.0051). Statistical analyses for the body length did not show any differences between the treatments at both temperatures (nested ANOVA: 7 °C: *p* = 0.0251 (with Dunnett’s Test: *p* > 0.05); 11 °C: *p* = 0.9296). In general, the brown trout larvae exposed to 11 °C had higher body weight and length than the larvae exposed at 7 °C.

#### Stress protein analyses

Neither the temperature nor metformin had an influence on the level of the stress protein Hsp70 in brown trout larvae (Table 1) (7 °C: nested ANOVA: *p *= 0.6287; 11 °C: Welch ANOVA: *p *= 0.1721).

#### Histological investigations

**Fig. 2:**
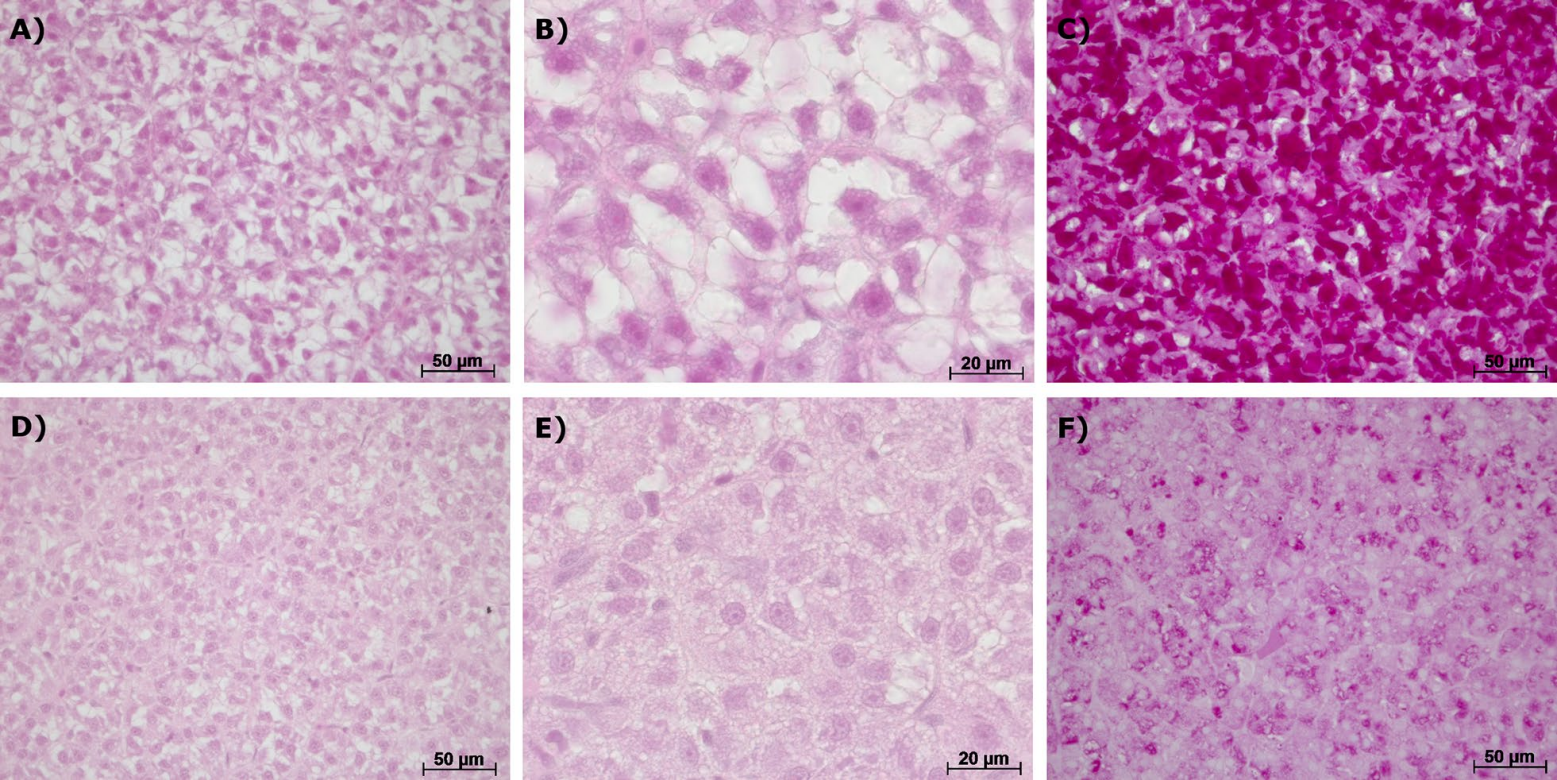
Representative sections of liver tissue of brown trout fry: **A**–**C**: control fish, **D**–**F**: fish exposed to 100 µg/L metformin. The controls showed large hepatocytes with high amounts of glycogen (stained with alcian blue-PAS in section **C**). Livers of fish exposed to metformin showed small hepatocytes with low glycogen amount (low staining intensity with alcian blue-PAS in section **F**). Sections **A**, **B**, **D** and **E** were stained with haematoxylin–eosin

In general, the liver tissue of the larvae were classified as category 1, 2 or 3 and did not show any strong histopathological alterations or severe damage of the tissue. In most livers, cells appeared large with a bright cytoplasm (Fig. 2A, B) containing high amounts of glycogen (Fig. 2C). Nevertheless, a number of individuals also had livers with small hepatocytes (Fig. 2D, E) and a low glycogen content (Fig. 2F). Small agglomerations of macrophages occurred in a few fish livers in all treatments, but further pathological changes could not be observed (further details in Additional file 1: Paragraph 6, Tables S11 and S12). The most prominent symptom was a modification of the glycogen content. Therefore, this parameter was crucial for the assessment values that have been allocated to the respective samples after semi-quantitative evaluation.

#### Histological and biochemical glycogen analyses

**Fig. 3:**
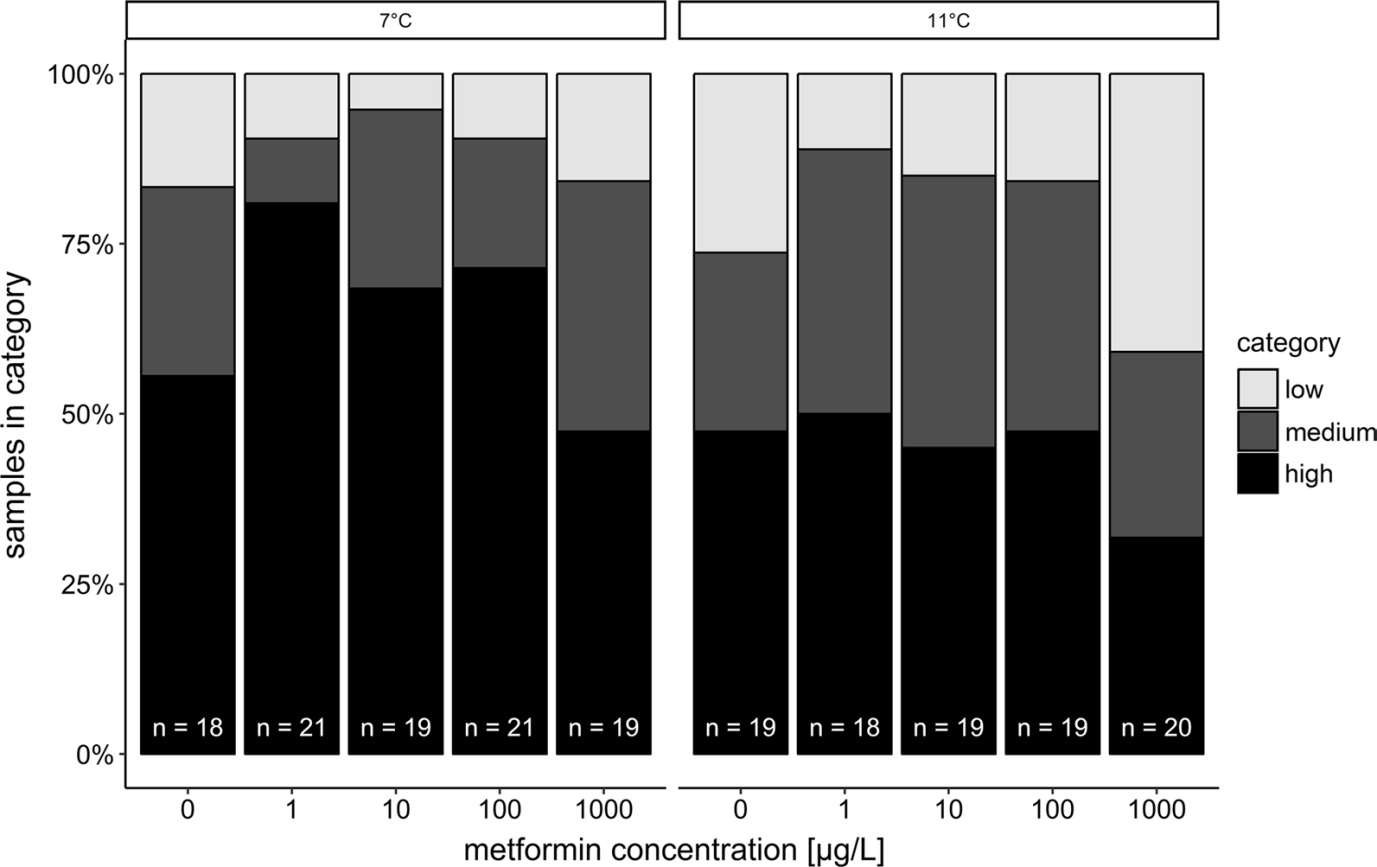
Semi-quantitative histological examination of the glycogen amount in the liver of brown trout larvae exposed to metformin at 7 °C and 11 °C categorised in low, medium and high glycogen contents (Likelihood-Ratio *χ*^2^ test: 7 °C: *p *= 0.5211; 11 °C: *p* = 0.3769). The number n of examined fish individuals is indicated in the bars

A qualitative comparison of the control individuals to determine whether temperature had an influence on the hepatic glycogen level revealed, for both histological examination and biochemical analysis, no difference between 7 and 11 °C. The histological examination showed that the liver glycogen amount was categorised as ‘high’ for 10 individuals, ‘medium’ for 5 and ‘low’ for 3 individuals at 7 °C. At 11 °C, there were 9 individuals with a glycogen content classified as ‘high’, 5 with a ‘medium’ glycogen amount and 3 with a ‘low’ one. Focussing solely on the effect of the drug, more individuals of the exposure groups with 1–100 µg/L metformin showed a hepatic glycogen content which was categorised as ‘high’ compared to the respective control group at 7 °C, especially for 1 µg/L (Fig. 3). At 11 °C, in contrast, such a relationship between metformin exposure and a ‘high’ glycogen content was not clear, but more individuals of the exposure groups with 1–100 µg/L metformin show a hepatic glycogen content which was categorised as ‘medium’ compared to the respective control group, again particularly at 1 µg/L; however, these differences were not significant (Likelihood-Ratio *χ*^2^ test: 7 °C: *p *= 0.5211; 11 °C: *p *= 0.3769).

**Fig. 4:**
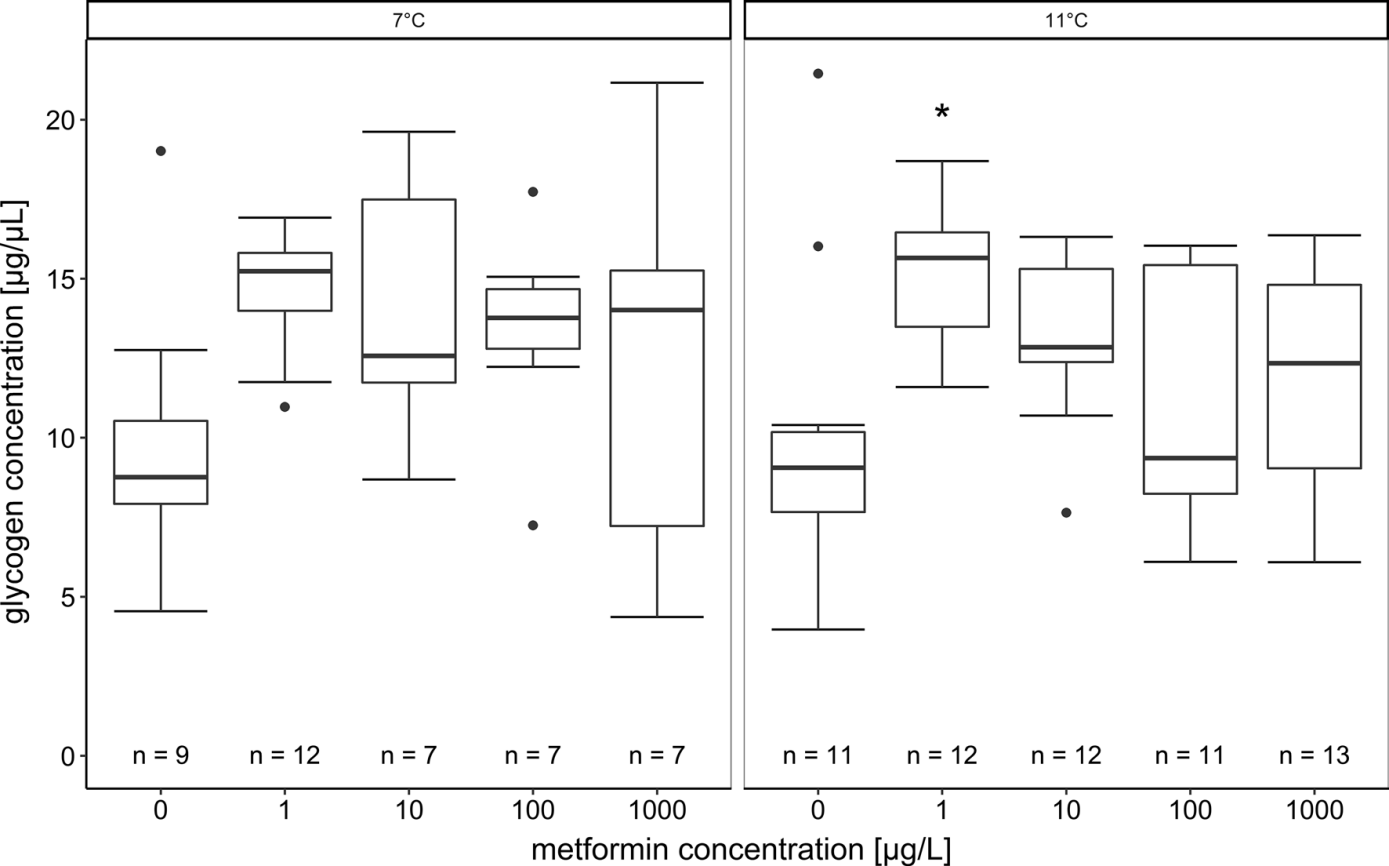
Hepatic glycogen content (measured with the biochemical glycogen assay) of brown trout fry exposed to metformin at 7 °C and 11 °C; the number n of examined fish individuals is indicated (reduced number of samples due to pre-tests and exclusion of values out of calibration curve). The line within the boxes represents the median, the boxes are bordered by the 25% to 75% quartiles, the whiskers show the minimum and maximum values, the black dots are outliers. The asterisk indicates a significant difference compared with the respective control at the *p* = 0.05 level (nested ANOVA: 7 °C: *p* = 0.0620; 11 °C: *p* = 0.0085 (with Dunnett’s Test: (0 µg/L|1 µg/L): *p* = 0.0024))

Also biochemical glycogen measurements (Fig. 4) revealed an increase of glycogen content in the livers of metformin-exposed larvae when compared to the control. At 11 °C, this difference was significant only for the lowest concentration, but the trend was also visible at 7 °C with a *p-*value slightly higher than 0.05 (nested ANOVA: 7 °C: *p *= 0.0620; 11 °C: *p *= 0.0085 (with Dunnett’s test: (0 µg/L|1 µg/L): *p *= 0.0024)).

For the intermediate concentrations of metformin (10 and 100 µg/L), the biochemical assay revealed an increased glycogen content compared to the controls; however, the effect was not as strong as for the lowest metformin concentration. In fish exposed to 11 °C, the glycogen content in the 100 µg/L metformin exposure group was distinctly lower compared to the 1 µg/L metformin treatment. However, at the highest concentration (1000 µg/L), the inter-individual variation of the glycogen amount within the exposure groups was rather high. The observed variation in glycogen content was visible with the histological and biochemical methods.

#### Behavioural analyses

The temperature of the test medium was shown to have an effect on the swimming behaviour of brown trout. Control fish that had not experienced metformin and were kept at 7 °C swam about 700 cm further in 18 min than control fish exposed at 11 °C. Also the velocity was about 0.6 cm/s higher at 7 °C compared to 11 °C. Concerning the effect of metformin solely, there was no difference of distance moved (7 °C: nested ANOVA: *p *= 0.9216; 11 °C: Welch ANOVA: *p *= 0.9690) and velocity (7 °C: nested ANOVA: *p *= 0.9215; 11 °C: Welch ANOVA: *p *= 0.9690) between the exposure groups, either at 7 °C or at 11 °C.

#### Microbiome analyses

**Fig. 5:**
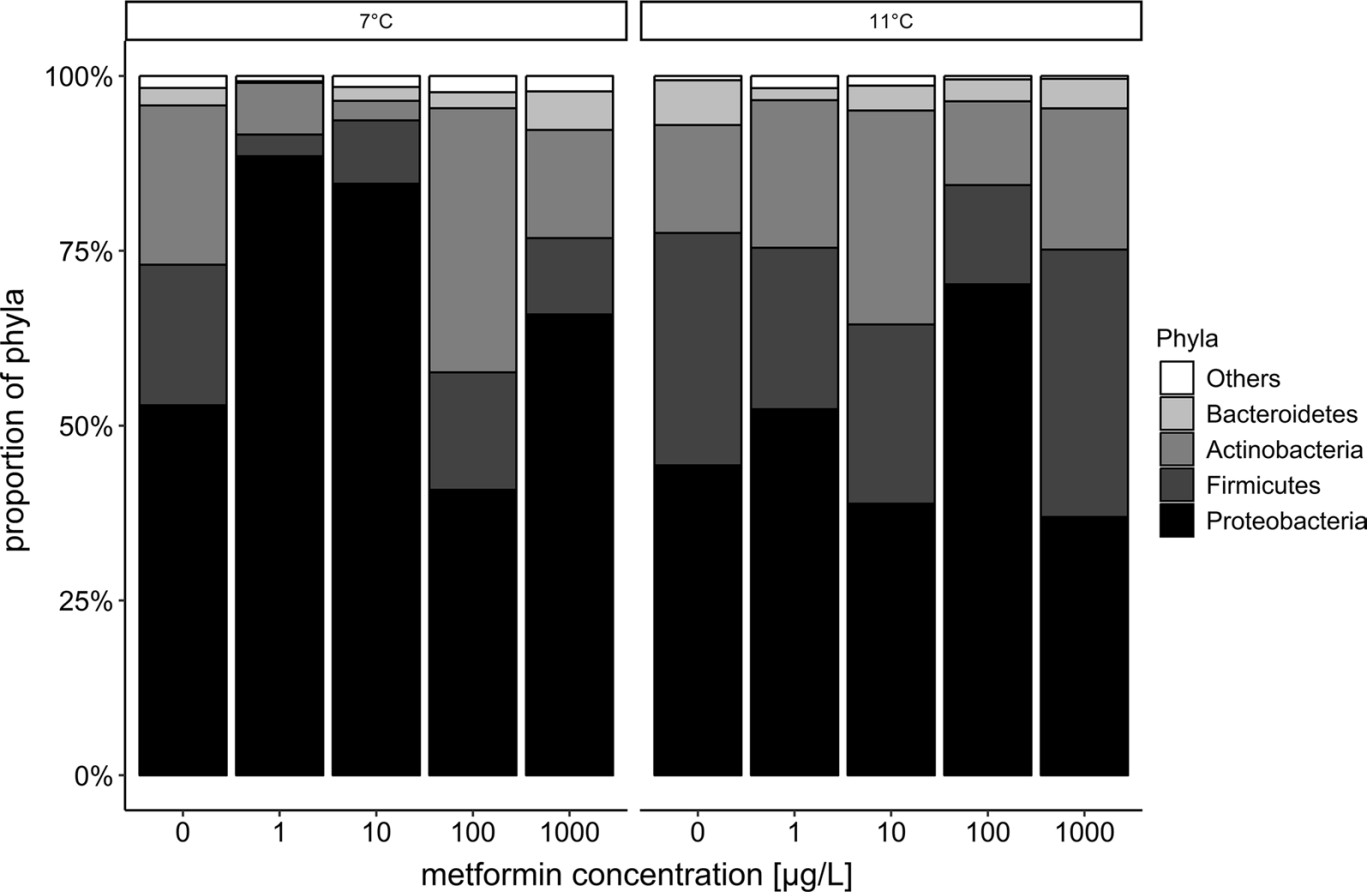
Composition of the intestinal microbiome of brown trout fry exposed to metformin at 7 °C and 11 °C. The analysis focuses on the four dominating phyla in the gut: Proteobacteria, Firmicutes, Actinobacteria and Bacteroidetes

The microbiome analysis includes 162290 sequences, classified into 773 operational taxonomic units (OTUs). In total, 98.7% of these sequences belonged to the four dominant phyla, Proteobacteria (63.6%), Firmicutes (15.2%), Actinobacteria (16.5%) and Bacteroidetes (3.4%) comprising a total of 705 OTUs. Keeping the low replicate number of samples in mind, fish exposed to 1 and 10 µg/L metformin at 7 °C and to 100 µg/L metformin at 11 °C seem to hold an intestinal microbiome with a different composition than that of the respective control fish: The proportion of the Proteobacteria in those individuals exposed to these metformin concentrations was found to be increased compared to the control, whereas the proportion of the Firmicutes and Actinobacteria was reduced (Fig. 5).

## Discussion

In the present study, the impact of the antidiabetic drug metformin on the development and health of brown trout larvae was investigated at 7 °C and 11 °C. The parameters focussing on development, such as heart rate and time to hatch, did not show any effects of metformin. Also mortality, stress protein levels, tissue integrity of the liver, body length and swimming behaviour were not influenced by metformin. However, an environmentally relevant concentration of 1 µg/L metformin led to a significantly enhanced liver glycogen content in brown trout larvae and a concentration range of 1–100 µg/L significantly decreased the body weight of the larvae. In the same concentration range, the investigation of the gut microbiome indicated a shift in the bacterial community. The observed metformin-induced changes did not follow a linear concentration effect relationship.

The chemical analysis of the larval tissue showed a concentration-dependent presence of metformin with temperature having an effect on the presence of the drug in the tissue. In particular for the highest metformin concentration, the tissue concentration of the drug was higher in fish exposed to 11 °C compared to the lower temperature which could be caused by an enhanced metabolism and uptake of metformin at higher temperatures. Several studies with mice and humans consuming metformin made it clear that the highest concentrations of metformin in the body can be found in the gastrointestinal tract, kidney and liver [7, 27, 52, 76]. Since the samples used for the chemical analysis only contained kidney, muscle and head without gills, but not liver or gut, the measured metformin concentrations in the tissue of our test organisms were most likely dominated by metformin in the kidney—assuming a similar distribution of metformin in fish as in human or mice. However, additional tissue samplings would have been necessary to determine whether the maximal tissue uptake capacity for metformin has already been reached at the end of the experiment. Also, one has to keep in mind that we could not show possible variations between the individual test organisms since the tissue samples were pooled per treatment.

In our study, metformin did not have any effect on mortality and the development of brown trout, since even sensitive endpoints such as the heart rate and mean time to hatch did not differ between the treatments. Consequently, metformin is not embryotoxic for brown trout. This assumption is supported by a report [19] cited by Moermond and Smit [45]. In this report, no effects of metformin on hatching rate, time to hatch and mortality in an early-life stage test with zebrafish could be displayed leading to a no observed effect concentration (NOEC) of ≥ 10 mg/L for metformin. Ussery [73] showed that metformin did not influence mortality, hatch success or time to hatch of Japanese medaka exposed to 1–100 µg/L metformin for 28 days. Likewise, an effect of the drug on the body length could not be observed in our experiment whereas the weight of brown trout larvae was found to be reduced when exposed to 10 and 100 µg/L metformin at 7 °C and 1 µg/L at 11 °C. This result reflects the use of the antidiabetic as a weight loss drug in human at doses of 1.5 to 2.5 g/day [40, 66]. As a potential mechanism responsible for metformin-induced weight loss in humans, the downregulation of appetite by attenuating the activity of the adenosine monophosphate-activated kinase (AMPK) in the hypothalamus has been proposed by Malin and Kashyap [40]. In the gastrointestinal tract, metformin increases the secretion of glucagon-like peptide 1 (GLP-1), a hormone which functions as satiation signal by decreasing the AMPK activity [40]. Moreover, two studies with fish also detected a reduction in weight at concentrations similar to those in our experiment. Niemuth and Klaper [50] showed that fathead-minnows weighed less when exposed to 40 µg/L metformin. Ussery [73] demonstrated that metformin decreased the weight and length of 28 days old Japanese medaka at concentrations of 1–100 µg/L. In contrast, in the assessment report mentioned above [19] the growth of zebrafish was not influenced by metformin.

The histopathological investigations revealed that the livers of the test organisms did not show pathological changes except for marginal macrophage agglomerations which occurred in a few animals only, but not consistently in a particular treatment. Thus, an effect of metformin on the tissue integrity of brown trout liver can be excluded.

It was expected that glycogen storage is lower at 11 °C than at 7 °C, as energy demands for maintenance and activity are increased at higher temperatures [74]. However, a qualitative comparison of the control individuals at the high and low temperatures did not reveal a difference in the hepatic glycogen content. Likewise, Barton and Schreck [6] showed that the glycogen content of juvenile Chinook salmon (*Oncorhynchus tshawytscha*) acclimated to 7.5 °C, 12.5 °C and 21.0 °C was reduced more at higher temperatures after being exposed to a stressor, but was restored to the same level in all temperature groups after a certain time. With respect to the influence of metformin on glycogen storage in the liver, the biochemical analysis showed that the lowest metformin concentration (1 µg/L) resulted in a high hepatic glycogen level and the histological examination indicated a similar trend. This finding can be explained by a limitation of metformin-triggered glycogenolysis which is one aspect of the therapeutic effect of this drug in humans [14, 25, 75]. With both methods, a high variability of the glycogen content in the livers of fish exposed to 1000 µg/L metformin became obvious. The low hepatic glycogen content in this exposure group could be caused by energy demanding processes induced by metformin resulting in a reduction of the liver glycogen or the reduced uptake of metformin in the liver so the drug could not fully exert its pharmaceutical effect. To date, we do not have a more detailed explanation for this finding.

The stress protein levels did not differ between the metformin-treated fish or between the controls exposed at the two temperatures. It is known that high base loads of stress proteins occur in developing organisms [38, 53] due to a high demand for the chaperones needed for correct protein folding. The high base level of Hsp70 in larvae compared to adult fish—as previously shown by Murtha and Keller [46] for *Danio rerio*—could conceal the effects of metformin, as described by Piro et al. [54], and temperature. To clarify whether or not metformin leads to a reduction in the stress protein level in brown trout, the Hsp70 level of older fish should be investigated. In a recent experiment conducted with 8-month-old brown trout, we were able to show that metformin did not influence the Hsp70 level, even in those fish (fish head was analysed, unpublished data, in Additional file 1: Paragraph 7, Fig. S1). Therefore, metformin probably does not have a reducing effect on the Hsp70 level in brown trout, but this cannot be excluded since we investigated the head in contrast to the pancreas, as in the study of Piro et al. [54].

The swimming behaviour of fish was not influenced by metformin. Although analyses regarding the energy status of the brown trout (hepatic glycogen content) showed a difference between the treatments, this effect was not reflected by behavioural parameters (distance moved and velocity). The study of Godoy et al. [26] also showed that the swimming behaviour of zebrafish was not influenced by metformin in comparable concentrations of 50 and 500 µg/L. Concerning the behavioural parameters, one can expect that animals exposed at higher temperatures move faster than those exposed at lower temperatures. The results for behaviour are not consistent with this expectation as we observed a higher activity of the larvae exposed at 7 °C. Since the EthoVision-system was installed in the climate chamber which was set to 11 °C, the higher ambient temperature during the record and the acclimatisation time could pose an additional stress factor and result in a higher swimming activity of these test organisms which were originally exposed at 7 °C.

Microbiological analyses indicated an effect of metformin on the intestinal microbiome of brown trout larvae, but this result must be verified by the analyses of the other replicates. Low to medium (1 and 10 µg/L at 7 °C and 100 µg/L at 11 °C) metformin concentrations led to a shift in the composition of the bacterial community of brown trout larvae which was also shown for mice and human [23, 37, 67, 77]. These effects of metformin on the microbiome are most likely caused by an accumulation of the drug in the intestine as shown by several studies [4, 27, 76]. In a parallel experiment conducted with 12-month-old brown trout, we were able to show that the amount of metformin per g tissue in the gut is about 3 to 60 times higher than in the kidney, liver or fillet (unpublished data, in Additional file 1: Paragraph 8, Fig. S2); however, it cannot be excluded that the measured metformin concentrations in the gut samples were influenced by metformin in the gut content, and not only determined by the presence of the drug in the tissue of the intestine. The different effect concentrations of the two temperatures could be explained with respect to the temperature preferences of the bacteria. Concerning the negative controls at the two temperatures, the proportion of Firmicutes was increased at 11 °C compared to 7 °C, whereas the proportion of Proteobacteria and Actinobacteria was decreased at 11 °C compared to 7 °C. Generally, a functional interpretation of the changes in the microbial community is not yet possible. Future analyses will focus on the presence of facultative-pathogenic bacteria with relevance for brown trout (e.g. Enterococci, Aeromonads) and the presence of species-specific virulence genes to determine whether and how the observed changes in the gut microbiome can affect the immune system and vitality of brown trout. Forslund et al. [23] have already described the increased expression of virulence factors and gas metabolism genes owing to metformin-associated shifts in the human gut microbiome. These shifts in the intestinal microbiome may have an indirect impact on the immune system [55].

Generally, the observed effects of metformin in brown trout concerning body weight, liver glycogen and the intestinal microbiome did not follow a linear concentration-effect relationship. However, a profound mechanistic explanation for this finding cannot be provided since the pharmaceutical action of the drug is still not fully elucidated. Scheen [60] and Graham et al. [27] indicated that metformin shows nonlinear pharmacokinetics caused by a reduced bioavailability at higher doses. Moreover, Chung et al. [11] revealed that effects on glycaemia did not linearly increase with higher doses of metformin in humans. The results of these studies led to the assumption that a similar phenomenon could have been possible in our study. Thus, metformin did not show an effect at the highest concentration. However, it would be favourable to complement our results of liver glycogen increase, the intestinal microbiome shift and body weight reduction and focus on the uptake of metformin at different concentrations in the identified target organs of the liver, intestine and brain to validate the assumptions for the nonlinearity of the obtained results.

Concerning the environmental relevance of the drug, it has to be stressed that other life stages of brown trout, e.g. adults and other species groups, e.g. invertebrates could react more sensitively to metformin, especially when exposed chronically. In addition to the effects on the metabolism of fish, several studies demonstrated endocrine effects of metformin such as vitellogenin mRNA upregulation or intersexes [13, 49–51] which can pose a risk for aquatic organisms and have to be included in the assessment of the environmental relevance of the drug. Apart from that, guanylurea—the main transformation product of metformin generated microbially in sewage-treatment plants—occurs at even higher concentrations than its parent compound in surface waters [61, 68]; the knowledge concerning its ecotoxicity is scarce, but would be required to judge the full ecotoxicological impact of metformin.

## Conclusions

Our study has made it clear that metformin did not induce lethal or embryotoxic effects in brown trout, and it became evident that it had no influence on the tissue integrity of the liver, on the stress protein level and on swimming behaviour or body length. However, metformin exerted effects related to the carbohydrate metabolism (changes of the liver glycogen), reduced body weight and influenced the gut microbiome already in low, environmentally relevant concentrations. These effects resemble the pharmaceutical effects of metformin in humans as antidiabetic and weight loss drug. To date, it is not known how the changes in the intestinal microbiome which occurred in the same concentration range can influence the vitality of the host organism, so further research is requested in this field.

The nonlinearity of the concentration–effect relation of the findings with the presence of effects only at low to medium metformin concentrations is also indicated in other studies, and this might be ascribed to the reduced bioavailability of metformin at higher doses. However, mechanistic explanations for this phenomenon are still lacking, and need to be studied further to fully understand metformin’s potential environmental impact.

## Additional file

**Additional file 1.** Additional figures and tables.

## Abbreviations

AcN: acetonitrile
AMPK: adenosine monophosphate-activated kinase
bpm: beats per minute
CE-MS: capillary electrophoresis-mass spectrometry
CRED: criteria for reporting and evaluation ecotoxicity data
DDD: defined daily doses
dpf: days post fertilisation
FA: formic acid
EMA: European medicines agency
GLP-1: glucagon-like peptide 1
Hsp: heat shock protein
LoQ: limit of quantification
MRM: multiple reaction monitoring
n.e.: not evaluated
NOEC: no observed effect concentration
OTUs: operational taxonomic units
PAS: periodic acid-Schiff
PCR: polymerase chain reaction
SD: standard deviation
