# Supplementary material for: Does the antidiabetic drug metformin affect embryo development and the health of brown trout (Salmo trutta f. fario)?
Source: Environ Sci Eur. 2018 Dec 7;30(1):48. doi: 10.1186/s12302-018-0179-4 (PMC6290717; doi:10.1186/s12302-018-0179-4)
Supplement: Supplementary file 1 — Additional file 1. Additional figures and tables. [file 12302_2018_179_MOESM1_ESM.docx]

**Impact of the antidiabetic drug metformin on embryo development of brown trout (*Salmo trutta* f. *fario*) and effects in developing larvae**

**Additional material**

## Chemical analyses

### Analysis of water concentrations by LC-MS

Tab. S1: Operating parameters of the triple quadrupole mass spectrometer (QqQ-MS)

| **Parameter** | **Set point** |
| --- | --- |
| Gas temperature | 150 °C |
| Gas flow | 16 L/min |
| Nebulizer | 45 psi |
| Sheath gas heater | 400 °C |
| Sheath gas flow | 12 L/min |
| Capillary voltage | 1500 V |
| Ion funnel high/low pressure RF | 90/70 V |
| Fragmentor voltage | 380 V |

Tab. S2: Operating parameters of the triple quadrupole mass spectrometer (QqQ-MS)

|  | Metformin | Metformin D6 |
| --- | --- | --- |
| Precursor ion (m/z) | 130 | 136 |
| Product ion (Quan/Qual) (m/z) | 60/71.1 | 60/77.1 |
| Collision energy (setpoint in V) | 10/30 | 10/30 |
| Cell accelerator voltage (V) | 6/6 | 6/6 |

Tab. S3: Intraday variations (RSD_r_) and interday variations (RSD_R_) of 1 µg/L standard solution measured by QqQ-MS

| RSD_r_ [%] | RSD_R_ [%) |
| --- | --- |
| (n=8) | (n=6) |
| 0.9 | 5.5 |

### Analysis of brown trout larvae and juvenile brown trout by CE-MS

We used a simple extraction procedure based on sonication with methanol for the determination of internal concentrations of metformin. Therefore, we homogenized frozen fish tissue with mortar and pestle under liquid nitrogen. Afterwards, three aliquots of 100 mg of the homogenized sample were weighed into a 2.5 mL tube (Eppendorf, Germany) and 50 µL deuterated internal standard metformin-D6 (Toronto Research Chemicals, Canada) were added, resulting in a final concentration of 291 nmol/L in the sample extract for injection. A volume of 440 µL methanol (Chromasolv, Sigma-Aldrich, USA) and 10 µL background electrolyte solution, consisting of 100 mM ammonium acetate (98 %) and 3 % acetic acid (100 %) (Merck Darmstadt, Germany) in methanol were added, and the sample was shaken for 30 s using a Vortex mixer. The analytes were extracted under sonication for 15 min and the precipitate was centrifuged at 13.000 g for 15 min. After filtration with 45 µm PTFE filter (pore size 0.45 μm, Chromafil Macherey-Nagel, Germany) the samples were analysed by CE-MS.

The CE separations were carried with an uncoated fused-silica capillary (length 80 cm, i.d. 50 µm). The samples were injected hydrodynamically by applying a pressure of 100 mbar for 10s. New capillaries were conditioned with 1 M NaOH for 15 min, water for 10 min and BGE for 15 min. Before each run the capillary was conditioned with BGE for 5 min. The CE capillary was kept at 25 °C during CE runs and a voltage of +30 kV was applied.

All analyses were performed using an Agilent CE 7100 interfaced to an Agilent 6550 iFunnel Q-TOF mass spectrometer (Agilent Technologies, Santa Clara, CA, USA) with an electrospray ionization source (ESI) assisted by sheath liquid interface. The composition of the sheath liquid was isopropanol/water (1:1, v/v) with 0.1 % FA. The sheath liquid was delivered by a 1260 isocratic pump (Agilent Technologies, Waldbronn, Germany) at a flow rate of 4 µL/min. The nebulizer pressure was set to 0.28 bar and the drying gas flow rate to 4 L/min. A fragmentor voltage of 175 V, a capillary voltage of ‑4.000 V, a skimmer voltage of 65 V and an octopole voltage of 750 V were used. The mass range was set to m/z 100–1.700, and the data acquisition rate was two spectra/s. For internal calibration purine, HP0321 and HP0921 (Agilent Technologies, Waldbronn, Germany) were used. Data analysis was accomplished using MassHunter software (Agilent Technologies, Waldbronn, Germany). Internal metformin concentrations were calculated based on peak area of the deuterated internal standard and the detection limit was 0.6 µg/L. The method precision is given in Table 4.

Tab. S4: Details for method precision of QqQ-MS-measurement of internal metformin concentrations of brown trout larvae. Given are the methodological variances for each treatment (two measurements of the pooled sample of each treatment) and both exposure temperatures.

|  | RSD [%] | |
| --- | --- | --- |
|  | (n=2) | |
| nominal metformin concentration in medium | 7 °C | 11 °C |
| 0 µg/L | 0 | 0 |
| 1 µg/L | 0 | 0 |
| 10 µg/L | 1.49 | 0.36 |
| 100 µg/L | 1.00 | 12.34 |
| 1000 µg/L | 0.60 | 21.66 |

## Histology: Alcianblue PAS staining

| **medium** | **duration** |
| --- | --- |
| Roti® -Histol | 3 min |
| 96% ethanol | 2 min |
| 80% ethanol | 2 min |
| 60% ethanol | 2 min |
| Aqua dest. | 5 min |
| 3% acetic acid | 3 min |
| alcianblue | 30 min |
| 3% acetic acid | 4 sec |
| Aqua dest. | 5 min |
| 1% perjodic acid | 10 min |
| Aqua dest. | 5 sec |
| Aqua dest. | 5 sec |
| Aqua dest. | 5 sec |
| Schiff | 5 min |
| sulfite water | 2 min |
| sulfite water | 2 min |
| sulfite water | 2 min |
| running tap water | 15 min |
| Aqua dest. | 5 sec |
| 70% ethanol | 5 min |
| 80% ethanol | 5 min |
| 90% ethanol | 5 min |
| 100% ethanol | 5 min |
| Roti® -Histol | 5 min |
|  | total: 1h 53 min |

## Water quality parameters

Tab. S5: Water quality parameters at the beginning of the experiment with brown trout larvae exposed to metformin

| Climate chamber | MF conc. [µg/L] | Replicate | Oxygen conc. [mg/L] | pH | Conductivity [µS/cm] | Temperature [°C] |
| --- | --- | --- | --- | --- | --- | --- |
| 7°C | 0 | 1 | 10.41 | 7.33 | 388 | 7.6 |
|  | 0 | 2 | 10.5 | 7.81 | 387 | 7 |
|  | 0 | 3 | 10.52 | 7.6 | 389 | 7 |
|  | 1 | 1 | 10.19 | 8.05 | 386 | 7.9 |
|  | 1 | 2 | 10.35 | 8.11 | 386 | 7.3 |
|  | 1 | 3 | 10.44 | 8.13 | 387 | 7.1 |
|  | 10 | 1 | 10.44 | 8.13 | 389 | 7.4 |
|  | 10 | 2 | 10.48 | 8.15 | 388 | 7 |
|  | 10 | 3 | 10.51 | 8.07 | 388 | 6.8 |
|  | 100 | 1 | 10.24 | 8.09 | 384 | 7.8 |
|  | 100 | 2 | 10.39 | 8.14 | 383 | 7.2 |
|  | 100 | 3 | 10.46 | 8.15 | 383 | 6.9 |
|  | 1000 | 1 | 10.42 | 8.11 | 425 | 7.4 |
|  | 1000 | 2 | 10.46 | 8.21 | 424 | 7 |
|  | 1000 | 3 | 10.5 | 8.22 | 424 | 6.9 |
| 11°C | 0 | 1 | 9.61 | 7.7 | 436 | 10.9 |
|  | 0 | 2 | 9.57 | 7.78 | 434 | 10.8 |
|  | 0 | 3 | 9.6 | 7.84 | 431 | 10.7 |
|  | 1 | 1 | 9.58 | 7.88 | 432 | 11 |
|  | 1 | 2 | 9.56 | 7.9 | 433 | 10.7 |
|  | 1 | 3 | 9.64 | 7.85 | 431 | 10.6 |
|  | 10 | 1 | 9.76 | 7.99 | 436 | 10.4 |
|  | 10 | 2 | 9.65 | 7.98 | 434 | 10.6 |
|  | 10 | 3 | 9.61 | 7.98 | 432 | 10.4 |
|  | 100 | 1 | 9.6 | 7.98 | 428 | 10.8 |
|  | 100 | 2 | 9.57 | 7.98 | 427 | 10.7 |
|  | 100 | 3 | 9.6 | 7.99 | 426 | 10.5 |
|  | 1000 | 1 | 9.73 | 8.08 | 431 | 10.4 |
|  | 1000 | 2 | 9.66 | 8.03 | 430 | 10.6 |
|  | 1000 | 3 | 9.62 | 8.01 | 428 | 10.6 |

Tab. S6: Water quality parameters at the day of the heart rate measurement of brown trout larvae in the control and exposed to 1000 µg/L metformin

| **Climate chamber** | MF conc. [µg/L] | Replicate | Oxygen conc. [mg/L] | pH | Conductivity [µS/cm] | Temperature [°C] |
| --- | --- | --- | --- | --- | --- | --- |
| 7 °C | 0 | 1 | 10.37 | 8.00 | 409 | 8.5 |
|  | 0 | 2 | 10.50 | 8.07 | 410 | 8.1 |
|  | 0 | 3 | 10.36 | 7.93 | 405 | 8.6 |
|  | 1000 | 1 | 10.26 | 8.04 | 408 | 8.1 |
|  | 1000 | 2 | 10.31 | 8.02 | 410 | 8.3 |
|  | 1000 | 3 | 10.19 | 7.90 | 408 | 8.4 |
| 11 °C | 0 | 1 | 9.10 | 7.09 | 423 | 12.6 |
|  | 0 | 2 | 9.38 | 7.40 | 423 | 11.7 |
|  | 0 | 3 | 9.52 | 7.68 | 423 | 11.1 |
|  | 1000 | 1 | 9.66 | 7.84 | 421 | 10.8 |
|  | 1000 | 2 | 9.66 | 7.85 | 421 | 10.9 |
|  | 1000 | 3 | 9.72 | 7.86 | 421 | 10.9 |

Tab. S7: Water quality parameters at the day of the heart rate measurement of brown trout larvae in the control and exposed to 1000 µg/L metformin

| **7 °C** |  | **0 µg/L metformin** | **1000 µg/L**  **metformin** |
| --- | --- | --- | --- |
| NO_3_^-^ | [mg/L] | 1.60 | 1.53 |
| NO_2_^-^ | [mg/L] | 0.01 | 0.01 |
| NH_4_^+^ | [mg/L] | 0.66 | 0.81 |
| SO_4_^2-^ | [mg/L] | 56.33 | 55.00 |
| total hardness | drop | 14.00 | 12.67 |
| total hardness | [mg/L CaCO_3_] | 249.20 | 225.47 |
| Carbonate hardness | drop | 9.67 | 8.33 |
| Carbonate hardness | [mg/L CaCO_3_] | 172.07 | 148.33 |
| Cl^-^ | [mg/L] | 17.67 | 16.67 |
| PO_4_^3-^ | [mg/L] | 0.22 | 0.18 |
|  | | | |
| **11 °C** |  | **0 µg/L metformin** | **1000 µg/L**  **metformin** |
| NO_3_^-^ | [mg/L] | 1.13 | 1.53 |
| NO_2_^-^ | [mg/L] | 0.06 | 0.10 |
| NH_4_^+^ | [mg/L] | 1.02 | 1.25 |
| SO_4_^2-^ | [mg/L] | 54.67 | 58.33 |
| total hardness | drop | 13.67 | 13.33 |
| total hardness | [mg/L CaCO_3_] | 243.27 | 237.33 |
| Carbonate hardness | drop | 8.67 | 8.33 |
| Carbonate hardness | [mg/L CaCO_3_] | 154.27 | 148.33 |
| Cl^-^ | [mg/L] | 17.67 | 18.00 |
| PO_4_^3-^ | [mg/L] | 0.23 | 0.63 |

Tab. S8: Water quality parameters at the end of the experiment with brown trout larvae exposed to metformin

| Climate chamber | MF conc. [µg/L] | Replicate | Oxygen conc. [mg/L] | pH | Conductivity [µS/cm] | Temperature [°C] |
| --- | --- | --- | --- | --- | --- | --- |
| 7°C | 0 | 1 | 10.41 | 6.99 | 437 | 7.4 |
|  | 0 | 2 | 10.64 | 7.25 | 438 | 6.8 |
|  | 0 | 3 | 10.74 | 7.9 | 440 | 7 |
|  | 1 | 1 | 10.36 | 8.15 | 422 | 7.4 |
|  | 1 | 2 | 10.74 | 8.19 | 425 | 6.8 |
|  | 1 | 3 | 10.8 | 8.21 | 427 | 7.1 |
|  | 10 | 1 | 10.81 | 8.27 | 425 | 7.1 |
|  | 10 | 2 | 10.78 | 8.26 | 417 | 7 |
|  | 10 | 3 | 10.72 | 8.24 | 419 | 7 |
|  | 100 | 1 | 10.51 | 8.32 | 419 | 7.8 |
|  | 100 | 2 | 10.65 | 8.34 | 422 | 7.1 |
|  | 100 | 3 | 10.69 | 8.34 | 420 | 7 |
|  | 1000 | 1 | 10.71 | 8.27 | 435 | 7.1 |
|  | 1000 | 2 | 10.73 | 8.28 | 432 | 6.9 |
|  | 1000 | 3 | 10.57 | 8.28 | 424 | 7.1 |
| 11°C | 0 | 1 | 9.97 | 8.01 | 451 | 10.6 |
|  | 0 | 2 | 9.9 | 8.03 | 441 | 10.8 |
|  | 0 | 3 | 9.9 | 8.05 | 439 | 10.7 |
|  | 1 | 1 | 9.81 | 8.06 | 414 | 11.1 |
|  | 1 | 2 | 9.88 | 8.06 | 432 | 10.8 |
|  | 1 | 3 | 9.9 | 8.05 | 440 | 10.8 |
|  | 10 | 1 | 10 | 8.18 | 421 | 10.8 |
|  | 10 | 2 | 9.87 | 8.17 | 420 | 11.2 |
|  | 10 | 3 | 9.85 | 8.17 | 427 | 10.6 |
|  | 100 | 1 | 9.85 | 8.16 | 414 | 11.1 |
|  | 100 | 2 | 9.92 | 8.16 | 424 | 10.7 |
|  | 100 | 3 | 9.92 | 8.18 | 417 | 10.6 |
|  | 1000 | 1 | 9.99 | 8.2 | 445 | 10.5 |
|  | 1000 | 2 | 9.72 | 8.16 | 442 | 11.3 |
|  | 1000 | 3 | 9.79 | 8.17 | 435 | 11 |

## Real water concentrations in the test aquaria

Tab. S9: Mean MF-water concentrations of the test aquaria per treatment for the experiment with brown trout larvae exposed to MF at 7°C, measured with LC-MS

| MF Treatment | beginning (05.01.16) | b. w.e. (15.01) | a. w.e. (15.01) | a. w.e. (15.02.) | a. w.e. (08.03.) | b. w.e. (24.03) | a. w.e. (24.03.) | a. w.e. (08.04) | end (21.04.16) |
| --- | --- | --- | --- | --- | --- | --- | --- | --- | --- |
| 0 µg/L | <LOD | <LOD | <LOD | <LOD | <LOD | <LOD | <LOD | <LOD | <LOD |
| 1 µg/l | 0.97 | 0.97 | 0.97 | 0.95 | 0.67 | 0.83 | 0.70 | 0.59 | 0.64 |
| 10 µg/l | 8.97 | 8.74 | 8.41 | 8.47 | 7.77 | 7.67 | 7.99 | 7.36 | 7.70 |
| 100 µg/L | 101.56 | 103.20 | 106.44 | n.m. | 94.75 | 110.97 | 100.86 | 74.89 | 83.53 |
| 1000 µg/L | 989.22 | 1003.70 | 999.75 | 888.83 | 884.37 | 1120.33 | 1062.90 | 749.50 | 859.25 |
| b.w.e.= before water exchange; a.w.e.= after water exchange; n.m. not measured | | | | | | | | | |

Tab. S10: Mean MF-water concentrations of the test aquaria per treatment for the experiment with brown trout larvae exposed to MF at 11°C, measured with LC-MS

| MF Treatment | beginning (05.01.16) | b. w.e. (15.01) | a. w.e. (15.01) | a. w.e. (15.02.) | a. w.e. (08.03.) | b. w.e. (24.03) | a. w.e. (24.03.) | end (08.04.18) |
| --- | --- | --- | --- | --- | --- | --- | --- | --- |
| 0 µg/L | <LOD | <LOD | <LOD | <LOD | <LOD | <LOD | <LOD | <LOD |
| 1 µg/l | 1.00 | 1.02 | 1.04 | 0.88 | 0.61 | 0.64 | 0.92 | 0.65 |
| 10 µg/l | 9.66 | 9.22 | 8.80 | 7.78 | 8.02 | 8.84 | 9.42 | 6.84 |
| 100 µg/L | 99.19 | 103.41 | 100.99 | 101.29 | 92.49 | 105.45 | 98.13 | 89.00 |
| 1000 µg/L | 1007.95 | 1008.10 | 1003.60 | 976.42 | 888.32 | 749.13 | 988.60 | 747.00 |
| b.w.e.= before water exchange; a.w.e.= after water exchange | | | | | | | | |

## Statistics

### Statistical output of the biochemical glycogen assay

Brown trout larvae (7°C) – Biochemical determination of the hepatic glycogen content – Nested ANOVA

| **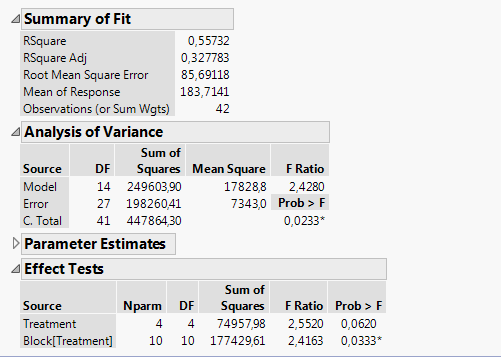** |
| --- |

Brown trout larvae (11°C) – Biochemical determination of hepatic glycogen content – Nested ANOVA with Post hoc-Dunnett’s Test

| **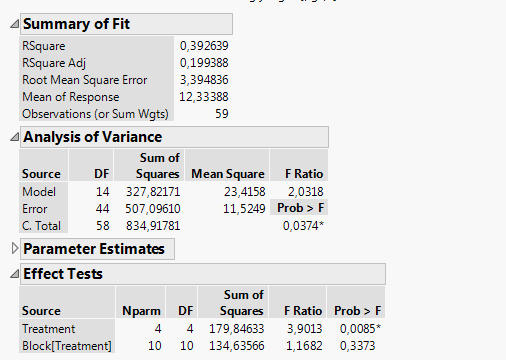** |
| --- |
| **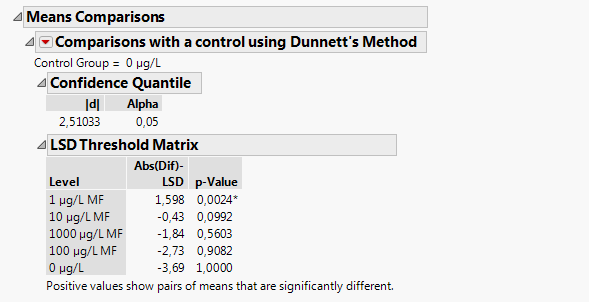** |

### Statistical output of the body weight analysis

Brown trout larvae (7°C) – Body weight – Nested ANOVA with Post hoc- Dunnett’s Test

| 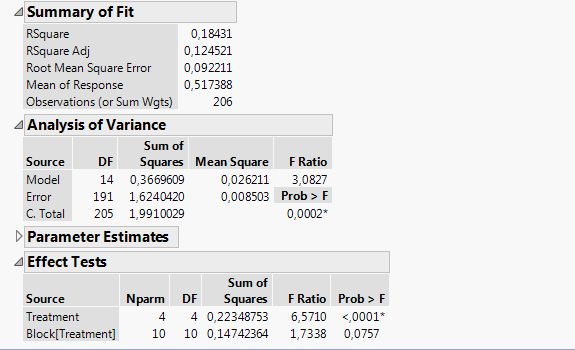 |
| --- |
| 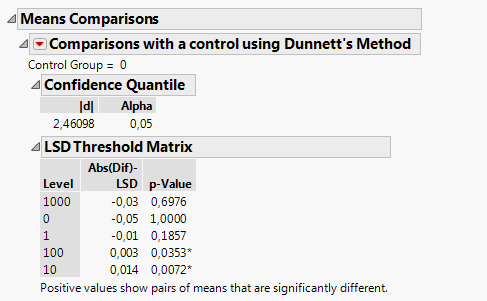 |

Brown trout larvae (11°C) – Body weight – Nested ANOVA with Post hoc-Dunnett’s Test

| 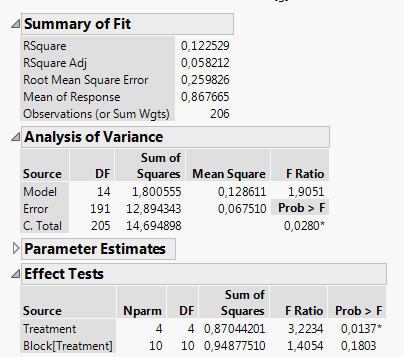 |
| --- |
| 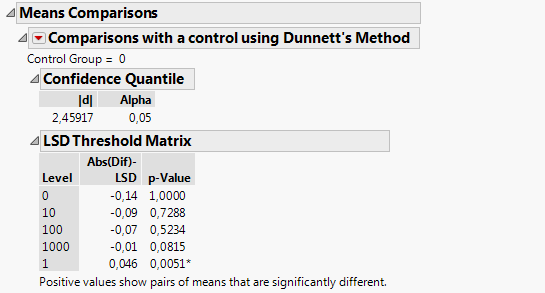 |

## Histopathological investigation of the liver of brown trout larvae

Supporting information for Tab. S11 and 12: qualitative examination of the samples: Histopathological findings in the liver of brown trout larvae

| **Dark grey** | **Grey** | **White** |
| --- | --- | --- |
| High glycogen content | Medium glycogen content | Low glycogen content |
| No macrophage agglomeration | Small macrophage agglomeration | Big macrophage agglomeration |
| Big cell size | Medium cell size | Small cell size |
| Bright cytoplasm | Medium cytoplasm | Dark cytoplasm |

Tab. S11: Detailed information for the histopathological findings of the liver of brown trout larvae exposed to metformin exposed at 7 °C (for the qualitative examination)

| **7°C** | **0 µg/L** | | | **1 µg/L** | | | **10 µg/L** | | | **100 µg/L** | | | **1000 µg/L** | | |
| --- | --- | --- | --- | --- | --- | --- | --- | --- | --- | --- | --- | --- | --- | --- | --- |
| Glycogen amount | 10 | 5 | 3 | 17 | 2 | 2 | 13 | 5 | 1 | 15 | 4 | 2 | 9 | 7 | 3 |
| Macrophage agglomeration | 17 | 1 | 0 | 20 | 1 | 0 | 19 | 0 | 0 | 19 | 2 | 0 | 17 | 2 | 0 |
| Cell size | 14 | 1 | 3 | 16 | 4 | 1 | 15 | 3 | 1 | 18 | 2 | 1 | 16 | 3 | 0 |
| Cytoplasm | 14 | 0 | 4 | 19 | 0 | 2 | 18 | 0 | 1 | 19 | 0 | 2 | 18 | 1 | 0 |

Tab. S12: Detailed information for the histopathological findings of the liver of brown trout larvae exposed to metformin exposed at 11 °C (for the qualitative examination)

| **11°C** | **0 µg/L** | | | **1 µg/L** | | | **10 µg/L** | | | **100 µg/L** | | | **1000 µg/L** | | |
| --- | --- | --- | --- | --- | --- | --- | --- | --- | --- | --- | --- | --- | --- | --- | --- |
| Glycogen amount | 9 | 5 | 5 | 9 | 7 | 2 | 9 | 8 | 2 | 9 | 7 | 3 | 7 | 5 | 7 |
| Macrophage agglomeration | 17 | 2 | 0 | 15 | 3 | 0 | 18 | 1 | 0 | 18 | 1 | 0 | 16 | 3 | 0 |
| Cell size | 9 | 7 | 3 | 14 | 2 | 2 | 10 | 8 | 1 | 13 | 3 | 3 | 9 | 7 | 3 |
| Cytoplasm | 11 | 0 | 8 | 17 | 0 | 1 | 17 | 1 | 1 | 15 | 0 | 4 | 12 | 3 | 5 |

## Stress protein analysis

Fig. S1: Analysis of the Hsp70-Level in the heads of juvenile brown trout (age: 8 month) exposed to metformin for 23 d (unpublished data)

## Chemical tissue analysis


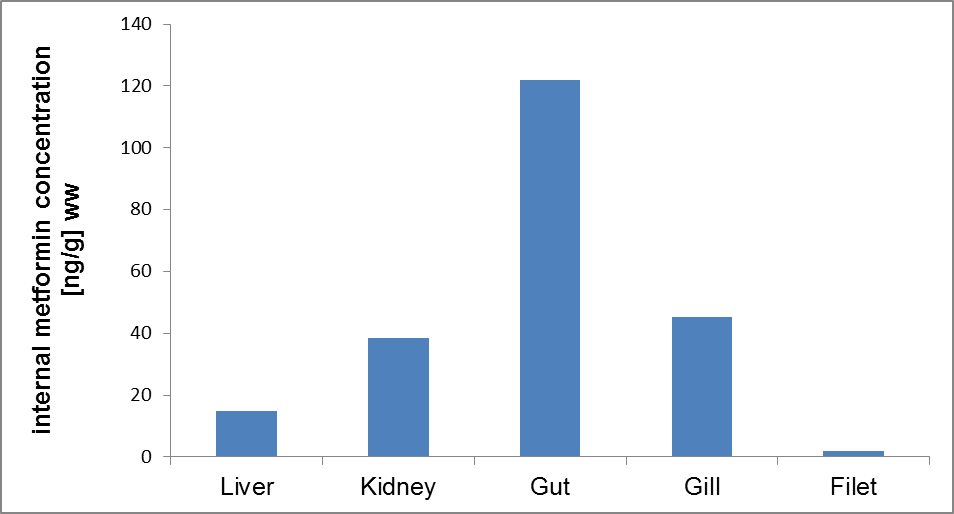


Fig. S2: Chemical analysis of metformin in different organs of brown trout (age: 12 month) exposed to metformin for 22 d (unpublished data)

## CRED-criteria

Tab. S13: Cred-Criteria according to Moermond, Kase et al. (2016) for the exposure experiment with brown trout larvae and metformin

| **CRED-criteria: Exposure of brown trout larvae to metformin** | | | | | |
| --- | --- | --- | --- | --- | --- |
| **1. General information** | | | | | |
| a. Purpose | | | Aim of the study was to investigate the effect of metformin on the health and development of brown trout larvae. | | |
| b. Endpoints | | | Histopathological investigation of the liver, biochemical determination of the hepatic glycogen content, analyses of the Hsp70-Level, of the behaviour, of the blood glucose level and the intestinal microbiome | | |
| **2. Test design** | | | | | |
| a. Standard | | | No standard test procedure | | |
| b. GLP | | | Not GLP-accredited | | |
| c. Controls | | | Laboratory negative control | | |
| d. Validity | | | The mortality of the control was <10%  (for 7°C: 2%, for 11°C: 3.34 %) | | |
| **3. Test compound** | | | | | |
| a. Identification | | 1,1-Dimethylbiguanide hydrochloride  CAS:1115-70-4. | | |  |
| b. Physico-chemical characteristics | | water solubility of 16.56 g/L (TOCRIS 2017) | | |  |
| c. Source | | Sigma-Aldrich, Product Number: D150959; Batch Number: BCBP0558V | | |  |
| d. Purity | | 99.9% according to the Certificate of Analysis from Sigma-Aldrich | | |  |
| e. Formulation | | no formulation, no impurities | | |  |
| **4. Test organism** | | | | |  |
| a. Scientific name | | *Salmo trutta* f. *fario* | | |  |
| b. Body weight/length | | 7°C: Body weight: mean=0.52 g ± 0.03  7°C: Body length: mean=3.50 cm ± 0.05  11°C: Body weight: mean=0.87 g ± 0.06  11°C: Body length: mean=4.22 cm ± 0.03 | | |  |
| c. Age/life stage | | From eyed egg stage until 8 weeks post yolk-sac consumption (Larvae) | | |  |
| d. Reproductive condition | | not in reproductive condition | | |  |
| e. Sex | | not determinable | | |  |
| f. Strain/clone | | no defined clone | | |  |
| g. Source | | commercial trout farm (Forellenzucht Lohmühle, D-72275 Alpirsbach-Ehlenbogen) | | |  |
| h. Acclimatisation | | No acclimatisation | | |  |
| **5. Exposure conditions** | | | | |  |
| a. Schedule | | semi-static design with water exchange of 50 % of the test medium twice a week | | |  |
| b. System | | closed | | |  |
| c. Test medium | | Filtered tap water (iron filter, active charcoal filter, particle filter) cooled to 7°C resp. 11°C and aerated | | |  |
| d. Temperature | | Climate chamber set to 7 °C resp. 11°C, measured at the beginning and the end of the experiment; for 7 °C: mean=7.16°C ± 0.30  for 11 °C: mean=10.74 ±0.23 | | |  |
| e. pH | | measured at the beginning and the end of the experiment;  for 7 °C: mean=8.05 ± 0.33  for 11 °C: mean=8.03 ± 0.13 | | |  |
| f. Hardness | | not measured | | |  |
| g. Conductivity | | measured at the beginning and the end of the experiment;  for 7°C: mean=410.43 µS/cm ± 20.29  for 11°C: mean=431.03 µS/cm ± 8.51 | | |  |
| h. Dissolved oxygen | | measured at the beginning and the end of the experiment;  for 7°C: mean=10.54 mg/L ± 0.16  for 11°C: mean=9.75 mg/L ± 0.15 | | |  |
| i. Light intensity/quality | | 10 h : 14 h light:dark cycle; aquaria shaded from direct light with black plastic foil | | |  |
| j. Feeding | | After yolk sac consumption, the brown trout were fed once per day with commerical trout feed (0.5 mm for the first 4 weeks post yolk sac consumption, then 0.8 mm (Inicio Plus, Biomar, Denmark)) | | |  |
| k. Aquaria | | 25 L glass aquaria filled with 10 L of medium, covered with glass plane, silicone tubing, aerated with airstones (JBL ProSilent Aeras Micro S2) | | |  |
| l. Sand/sediment | | no sediment tested | | |  |
| m. Stock solutions | | Stock solution 1 (100 mg/L) prepared from 128.23 mg metformin hydrochloride in 1 dest. water, stock solution 2 (10 mg/L) produced from stock solution 1 via 1:10 dilution | | |  |
| n. Nominal concentrations | | 0, 1, 10, 100, 1000 μg/L | | |  |
| o. Measured concentration | | Water samples were taken and analysed at the beginning and the end of the experiment as well as at two times during the experiments, before and after the water exchange. mean: < LoD, 9.7, 889.7 μg/L | | |  |
| p. Method | | HPLC-MS (QqQ-MS) (LoQ = 1 ng/L) | | |  |
| q. Duration | | For 7°C: (05.01.16 – 21.04.16)  For 11°C: (05.01.16 – 08.04.16) | | |  |
| r. Observations | | Mortality, behaviour, at the end of the experiment body length and body weight were determined and samples were taken for biochemical and histological analyses | | |  |
| s. Results | | summary table in article | | |  |
| t. Biomass loading | | For 7°C: mean= 0.73 g/L  For 11°C: mean=1.22 g/L | | |  |
| **6. Statistical Design and Biological Response** | | | |  |  |
| a. Replicates | Three replicate aquaria per test concentration and temperature | | |  |  |
| b. Number of organisms | 28 fish per replicate | | |  |  |
| c. Design | In each climate chamber: three blocks, one replicate per treatment present in each block, arranged in randomized order | | |  |  |
| d. Statistical methods | Mortality and hatching rate: nested COX-regression, body length/mass, heart rate, stress proteins, swimming behaviour, blood glucose level: nested ANOVA, histology: Likelihood ratio χ^2^-test | | |  |  |
| e. Biological response | 1.) Increased hepatic glycogen amount at 1 µg/L MF ( significant at 11°C, clear trend at 7°C)  2.) Increased body weight at 10 and 100 µg/L MF (at 7°C) and at 1 µg/L MF( at 11°C) respectively | | |  |  |
| f. Dose-response | Dose-response not observed | | |  |  |
| g. Statistical significances | Increased hepatic glycogen amount at 1 µg/L MF (at 11°C)  Increased body weight at 10 and 100 µg/L MF (at 7°C) and at 1 µg/L MF( at 11°C) respectively | | |  |  |
| h. Significance level | α = 0.05, in cases of multiple comparisons adjusted via sequential Bonferroni | | |  |  |
| i. Variability | not estimated | | |  |  |
| j. Raw data | provided on request | | |  |  |

## Number of examined fish individuals


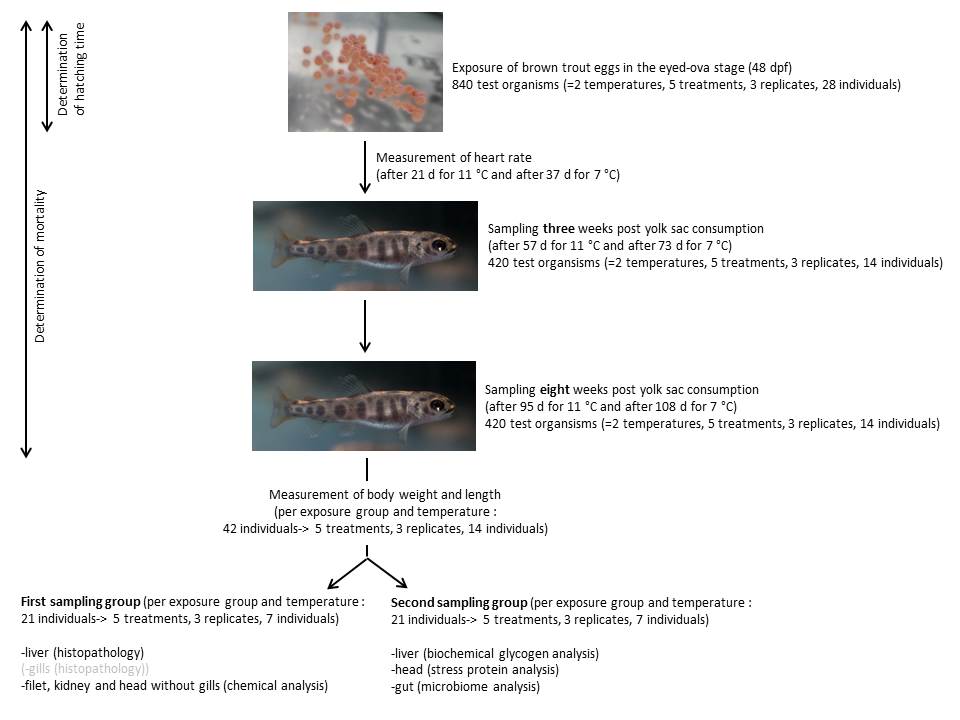


Fig. S3: Overview of the experiment with brown trout larvae and metformin. Given are the different sampling times and the number of individuals used.

Tab. S14: Number of examined fish individuals for body weight & length, stress protein level, histological and biochemical determination of the liver glycogen content and the behavioural parameters

| Parameter | 7 °C | | | | | 11 °C | | | | |
| --- | --- | --- | --- | --- | --- | --- | --- | --- | --- | --- |
|  | 0 µg/L | 1 µg/L | 10 µg/L | 100 µg/L | 1000 µg/L | 0 µg/L | 1 µg/L | 10 µg/L | 100 µg/L | 1000 µg/L |
| body weight | 41 | 41 | 41 | 42 | 41 | 40 | 41 | 40 | 41 | 44 |
| body length | 41 | 42 | 41 | 42 | 41 | 40 | 41 | 40 | 41 | 44 |
| Hsp70 | 20 | 20 | 21 | 21 | 20 | 19 | 20 | 19 | 20 | 20 |
| histopathological examination and histological determination of hepatic glycogen | 18 | 21 | 19 | 21 | 19 | 19 | 18 | 19 | 19 | 20 |
| biochemical determination of hepatic glycogen | 9 | 12 | 7 | 7 | 7 | 11 | 12 | 12 | 11 | 13 |
|  | 21 samples per exposure group  -> reduced number of samples due to pre-tests to find the optimal dilution  ->exclusion of samples if they did not lie in the calibration curve (high variability of the samples) | | | | | 21 samples per exposure group  -> reduced number of samples due to pre-tests to find the optimal dilution  ->exclusion of samples if they did not lie in the calibration curve (high variability of the samples) | | | | |
| total distance moved | 15 | 15 | 15 | 15 | 15 | 15 | 15 | 15 | 15 | 15 |
|  | 15 fish for swimming behaviour  ->5 fish per exposure group and replicate were used | | | | | 15 fish for swimming behaviour  ->5 fish per exposure group and replicate were used | | | | |
| mean velocity | 15 | 15 | 15 | 15 | 15 | 15 | 15 | 15 | 15 | 15 |
|  | 15 fish for swimming behaviour  ->5 fish per exposure group and replicate were used | | | | | 15 fish for swimming behaviour  ->5 fish per exposure group and replicate were used | | | | |
| Intestinal microbiome analysis | 21 gut samples per exposure group  (3 replicates, 7 individuals)  -> subsamples: the guts of 3 individuals per replicate were pooled  -> analysed is 1 out of 3 subsamples | | | | | 21 gut samples per exposure group  (3 replicates, 7 individuals)  -> subsamples: the guts of 3 individuals per replicate were pooled  -> analysed are 2 out of 3 subsamples | | | | |
| Chemical tissue analysis | 21 tissue samples per exposure group  (3 replicates, 7 individuals)  -> subsamples: the tissue samples of all 21 individuals per exposure group were pooled and divided in 2 subsamples | | | | | 21 tissue samples per exposure group  (3 replicates, 7 individuals)  -> subsamples: the tissue samples of all 21 individuals per exposure group were pooled and divided in 2 subsamples | | | | |

## Statistical details

Tab. S15: Details for the statistical analyses of the experiments with brown trout larvae exposed to metformin

| Investigated Parameter | Detailed statistical information |
| --- | --- |
| Mortality | 7 °C: COX-regression: df=4, χ^2^=0, p=1.0000  11 °C: COX-regression: df=4, χ^2^=0.0057, p=1.0000 |
| Mean time to hatch | 7 °C: COX-regression: df=4, χ^2^=3.3782, p=0.4966  11 °C: COX-regression: df=4, χ^2^=2.838, p=0.5853 |
| Heart rate | 7 °C: nested ANOVA: df_1_=1, df_2_=24, F=2.1127, p=0.1590;  11 °C: nested ANOVA: df_1_=1, df_2_=24, F=0.2975, p=0.5905 |
| Body weight | 7 °C: nested ANOVA: df_1_=4, df_2_=191, F=6.5710, p<0.0001; Dunnett’s test: (0 µg/L\|10 µg/L): p= 0.0072, (0 µg/L\|100 µg/L): p= 0.0353  11 °C: nested ANOVA: df_1_=4, df_2_=191, F=3.2234, p=0.0137; Dunnett’s Test: (0 µg/L\|1 µg/L): p= 0.0051 |
| Body length | 7 °C: nested ANOVA: df_1_=4, df_2_=191, F=2.8520, p=0.0251, Dunnett’s Test: p> 0.05;  11 °C: nested ANOVA: df_1_=4, df_2_=191, F=0.2156, p=0.9296 |
| Stress protein level (Hsp70) | 7 °C: nested ANOVA: df_1_=4, df_2_=88, F=0.6496, p=0.6287;  11 °C: Welch ANOVA: df numerator=4 /df denominator= 45.815, p=0.1721, squared for transformation |
| Histological glycogen determination | 7 °C: Likelihood-Ratio χ^2^ test: dF=8, χ^2^=7.144, p=0.5211;  11 °C: Likelihood-Ratio χ^2^ test: df=8, χ^2^=8.603, p=0.3769 |
| Biochemical glycogen determination | 7 °C: nested ANOVA: df_1_=4, df_2_=27, F=2.5520, p=0.0620, squared for transformation;  11 °C: nested ANOVA: df_1_=4, df_2_=44, F=3.9013, p=0.0085; Dunnett’s Test: (0 µg/L\|1 µg/L): p=0.0024). |
| Total distance moved | 7 °C: nested ANOVA: df_1_=4, df_2_=60, F=0.2281, p=0.9216;  11 °C: Welch-ANOVA: df numerator=4 /df denominator=34.445, p=0.9690 |
| Mean velocity | 7 °C: nested ANOVA: df_1_=4, df_2_=60, F=0,2282, p=0.9215;  11 °C: nested ANOVA: df numerator=4 /df denominator=34.445, p=0. 0.9690 |
